# Effect of Missing-Linker Defects and Ion Exchange on Stability and Proton Conduction of a Sulfonated Layered Zr-MOF

**DOI:** 10.1021/acsami.3c03873

**Published:** 2023-06-02

**Authors:** Monika Szufla, Jorge A. R. Navarro, Kinga Góra-Marek, Dariusz Matoga

**Affiliations:** † Faculty of Chemistry, Jagiellonian University, Gronostajowa 2, 30-387 Kraków, Poland; ‡ Doctoral School of Exact and Natural Sciences, Jagiellonian University in Kraków, Gronostajowa 2, 30-387 Kraków, Poland; § Departamento de Química Inorgánica, Universidad de Granada, 18071 Granada, Spain

**Keywords:** metal−organic frameworks, defect engineering, zirconium, proton transport, adsorption

## Abstract

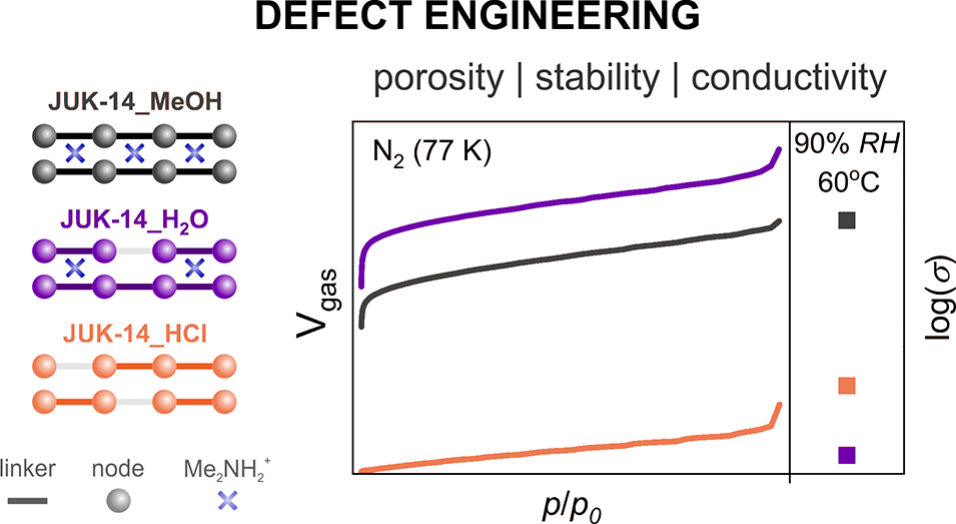

Intentionally introduced defects into solid materials create opportunities to control and tune their diverse physicochemical properties. Despite the growing interest in defect-engineered metal–organic frameworks (MOFs), there are still only a handful of studies on defective proton-conducting MOFs, including no reports on two-dimensional ones. Ion-conducting materials are fundamentally of great importance to the development of energy storage and conversion devices, including fuel cells and batteries. In this work, we demonstrate the introduction of missing-linker defects into a sulfonated proton conductive 2D zirconium-based MOF (**JUK-14**), using a facile post-synthetic approach and compare the stability and performance of the resulting materials, including proton conductivity, as well as adsorption of N_2_, CO_2_, and H_2_O molecules. We also discuss the associated presence of interlayer counterions and their effect on the properties and stability. Our approach to defect engineering can be extended to other layered MOFs and used for tuning their activity.

## Introduction

For many years after the first metal–organic framework (MOF) was synthesized and its crystal structure was determined, there was a belief that MOFs were “ideal” materials. However, systematic, in-depth investigations into the nature of these porous materials have shown that even seemingly ideal structures can contain a certain kind of disorder. In 2015 “*sites that locally break the regular periodic arrangement of atoms or ions of the static crystalline parent framework because of missing or dislocated atoms or ions*” were defined as defects in MOFs/coordination networks.^1^ Over the past decade, the percentage that defective MOFs account for in the total MOF publications has increased tenfold from 0.43 in 2012 to 4.3% in 2022 (*Web of Science* database search using the keyword strings “metal–organic frameworks” and “defect metal–organic frameworks”). Nowadays, the existence of defects in metal–organic frameworks is not only a challenge but also an opportunity to functionalize MOF materials, whereby a variety of methods for introducing defects have been developed, including both *de novo* synthesis and post-synthetic modifications.^2,3^ The missing-linker defects are one of the most common types of defects, featuring the absence of a part of the organic linkers. In place of the missing linker, coordination unsaturated sites (CUS) appear on the metal cluster, which can provide both access to the metal center and a platform for further modification.^4^ Materials with such defects, due to the presence of Lewis-type acidity and an improved sorption capacity, can be applied in catalysis,^5^ decontamination,^6^ dyes absorption and degradation,^7^ sorption,^8^ drug delivery,^9^ sensing,^10^ as well as charge transport, including ionic conductivity.^11−13^

Ion-conducting materials are fundamentally of great importance to the development of energy storage and conversion devices, including fuel cells and batteries. While there is a growing number of papers concerning defective MOFs, those characterizing the relationship between defects and proton conductivity are only a few.^4,14−17^ All these described defective proton-conducting MOFs have a zirconium cluster as a network node, and defects in the materials are created at the synthesis stage. All materials are also three-dimensional (3D), but they differ in connectivity (UiO-66 and UiO-66-SO_3_H are 12-connected, while MOF-808 is 6-connected) and ligand nature (only UiO-66-SO_3_H has a hydrophilic linker; ESI, Table S1). Connectivity lower than 12 results in the presence of terminal OH and H_2_O ligands already in ideal frameworks (counterparts without missing-linker defects), improving their overall hydrophilicity, which is an important parameter for proton conductivity. Considering all the cases mentioned, however, it is still not possible to determine the direct dependence of proton conductivity on defects. Many aspects need to be taken into account, such as the type of linker, the way defects are formed, their number and distribution, the saturation of defect sites, and the mobility of protons. The stability of the frameworks is also of great importance, since too many defects can destroy the network continuity and thus hinder efficient proton transport.^4^

The whole topic of defects among zirconium-based MOFs (Zr-MOFs), known for their high stability, is dominated by the 3D material UiO-66.^18^ To the best of our knowledge, there is only one case of studying defects in 2D Zr-MOFs.^19^ In this regard, it should be noted that 2D MOFs, compared to 3D materials, often exhibit very different properties and modification possibilities.^20^ Along with the general paucity of research on defective proton-conducting MOFs, this prompted us to investigate the first 2D zirconium-based MOF with defects for proton conduction. In this work, we demonstrate post-synthetic modification of a sulfonated proton-conducting 2D Zr-MOF (**JUK-14**), involving incorporation of missing-linker defects and removal of interlayer counterions, as well as we present an analysis of the impact of these modifications on the stability and performance of the resulting materials, particularly on the proton conduction.

## Results and Discussion

### Synthesis and Structure of JUK-14 Derivatives

**Figure 1:**
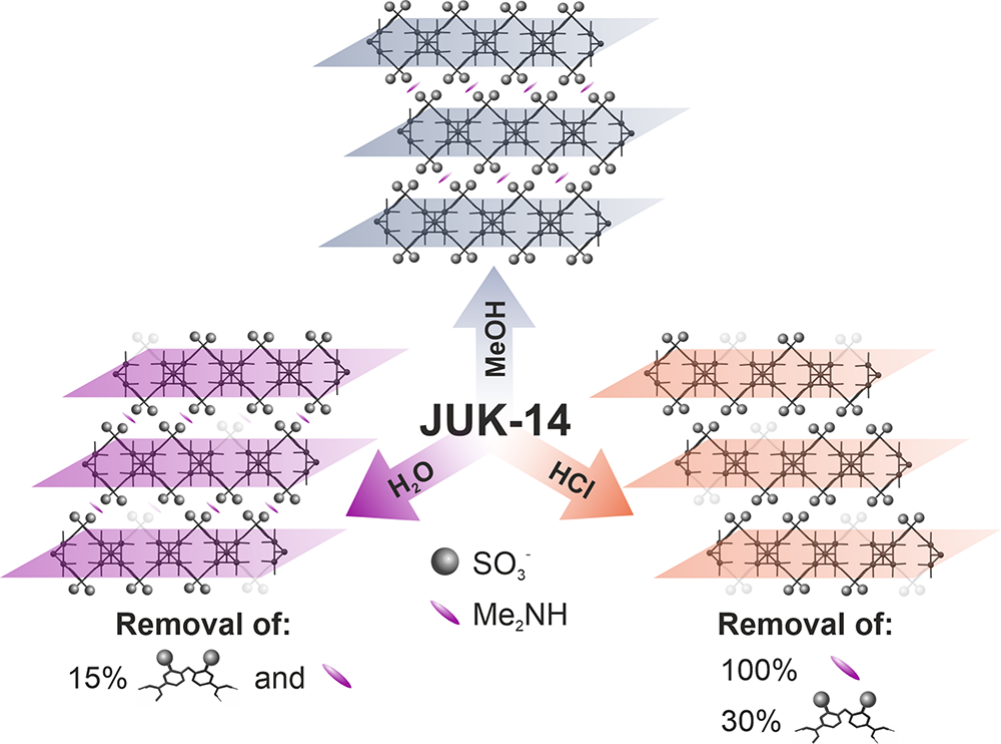
Schematic representation of the formation of defective **JUK-14** materials. Missing linkers are visualized as pale structural fragments.

**JUK-14** is a layered zirconium-based MOF containing Zr_6_O_4_(OH)_4_ oxoclusters bridged by angular ditopic linkers (dsoa-^4–^ - 4,4′-oxybis[3-(sulfonate)benzoate]; ESI, Figure S2) containing two sulfonate groups. In the unmodified structure, the negative charge of the linkers is compensated by dimethylammonium counterions.^21^ We have recently determined the crystal structure of **JUK-14** by single-crystal X-ray diffraction (CSD Number 2149732) with the guest-free formula (Me_2_NH_2_)_8_[Zr_6_(μ_3_-O)_4_(μ_3_-OH)_4_(μ-dsoa)_4_(OH)_4_(H_2_O)_4_]_*n*_, and described its proton conducting properties. In this work, we decided to investigate the possibility of introducing defects into this MOF in order to modulate its properties. To this end, the as-synthesized **JUK-14** (ESI, Figure S3) was immersed in three liquids: methanol, distilled water, and 1 M hydrochloric acid. As a result, three variants of **JUK-14** differing in the number and type of defects were obtained: a near-perfect **JUK-14_MeOH** after guest replacement only, **JUK-14_H_2_O** with missing linkers, and **JUK-14_HCl** with a higher amount of missing linkers (as compared to **JUK-14_H_2_O**) and without interlayer dimethylammonium cations (Figure 1 and ESI, Figure S4).

#### IR, NMR, and PXRD characterization

**Figure 2:**
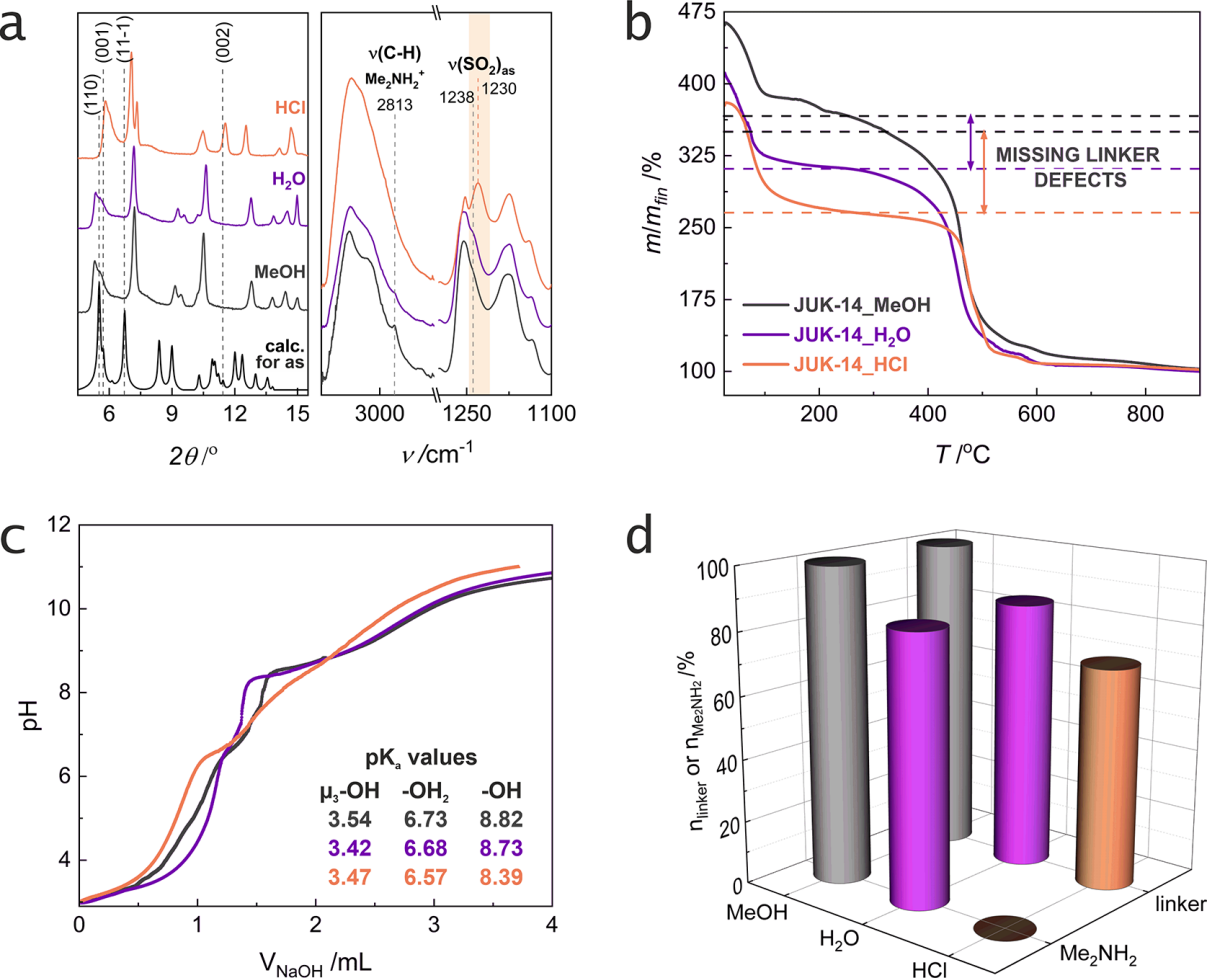
Evaluation of solids after the immersion of **JUK-14** in MeOH, water, and 1 M HCl: (a) PXRD patterns (left) and IR spectra (right); the calculated pattern for the as-synthesized **JUK-14** is added for comparison (black). (b) TGA curves. (c) Acid–base titration curves with calculated p*K*_a_ values. (d) Amounts of linkers and dimethylammonium ions are given as percentages (4 linkers or 4 ions per Zr_6_ cluster are assumed as 100%). The color code from Figure 2b refers to all plots (**JUK-14** immersed in MeOH – gray, H_2_O – violet, and HCl – orange).

In the earlier work on **JUK-14**, it was shown that the exchange of guest molecules from DMF to MeOH or H_2_O induces a “wine-rack” motion of the framework, resulting in a change in the position of the diffraction peaks, corresponding to the (110) and (11–1) lattice planes, which is also observed in this case (Figure 2a and ESI, Figures S9, S10). Powder X-ray diffraction (PXRD) patterns and IR spectra for **JUK-14_MeOH** and **JUK-14_H_2_O** (Figure 2a and ESI, Figures S9, S10) do not reveal any differences, which suggests that long-range order is essentially the same in these frameworks. On the other hand, the PXRD pattern of **JUK-14_HCl** is different, indicating that there is a structural change upon the transition from **JUK-14** to **JUK-14_HCl**. Complementary insight from IR spectroscopy shows the absence of the characteristic C–H stretch of dimethylammonium cations (at 2813 cm^–1^) for **JUK-14_HCl**, as well as a shift of the band corresponding to the asymmetric vibration of the SO_2_ group. The NMR spectra also corroborate the complete removal of dimethylammonium cations from the **JUK-14_HCl** material and further indicate the presence of 8 cations (per Zr_6_ cluster, that is one Me_2_NH_2_^+^ per SO_3_^–^ group) in **JUK-14_MeOH** and one Me_2_NH_2_^+^ per two SO_3_^–^ moieties in **JUK-14_H_2_O** (ESI, Figure S5). This shows that there are not 4 (as we formerly assumed^21^) but 8 dimethylammonium cations in the **JUK-14** material, where half of them are strongly bound to the framework and half considerably weaker and therefore can be removed much more readily (e.g., upon immersion of this material in water). Collectively, these observations confirm the complete removal of interlayer Me_2_NH_2_^+^ ions, including those strongly hydrogen-bonded to sulfonate groups, from **JUK-14_HCl**. This elimination is responsible for the changes in the PXRD pattern of the **JUK-14_HCl** material.

#### Thermogravimetric Analysis

Information about the presence of defects and the estimation of their amount was provided by thermogravimetric (TG) analysis (Figure 2b and ESI, Figures S6–S8, S11). For all **JUK-14** derivatives, the initial mass loss observed in the TG curves up to around 100 °C, is associated with the removal of guest water molecules. In addition, at around 200 °C, a second step is noticeable for **JUK-14_MeOH**, associated with the removal of dimethylamine coming from the weakly bound dimethylamonium cations (half of their initial amount). The plateau present between 150 and 400 °C refers to the MOF backbone itself, that is, the guest-free material. Subsequently, the highest mass loss between 450 and 600 °C is related to the decomposition of the framework and the combustion of the organic linker in the air. The final stage reflects the formation of a residual inorganic product. Assuming that the product of a Zr-based MOF decomposition in air is ZrO_2_, the theoretical position of the plateau, corresponding to the mass of the guest-free framework, was calculated (gray dashed lines in Figure 2b). Then, by comparing this theoretical plateau with the experimental one, the actual number of linkers in the analyzed material was determined (the detailed calculation method is given in ESI and Figure S1). Based on the most pronounced, yet imperfect plateau, the temperature of 250 °C was accepted as the reference for all guest-free frameworks. For **JUK-14_MeOH**, the number of linkers per Zr_6_ cluster determined by this method is slightly overestimated as 4.3 due to the different TG profile with a steep slope, and arbitrarily assumed to be 4.0 (Figure 2b and ESI, Figure S6). As mentioned above, the TG profile for **JUK-14_MeOH** indicates the removal of part of the dimethylammonium cations (an additional step on the TG curve at approximately 200 °C). Furthermore, this conclusion is supported by the thermogravimetric curve and elemental analysis of the **JUK-14_MeOH** material subjected to a series of modifications (reflecting the conditions of impedance measurements), such as activation in 100 °C, 10 mbar, conditioning in 90% RH, 60 °C, and soaking in MeOH, where the TG profile does not show a step associated with the removal of cations, and the EA indicates a significant reduction in nitrogen content (ESI, Figure S6 and Table S2). This also demonstrates that, although the material after soaking in methanol still has 8 Me_2_NH_2_^+^, when subjected to modifications, such as activation at elevated temperatures and reduced pressure and/or conditioning in high humidity, it loses half of the cations. Therefore, we can conclude that the material subjected to the adsorption and impedance experiments (described below) has 4 cations per Zr_6_ cluster. For **JUK-14** soaked in water, we carried out an equivalent modification to that for methanol soaking, but due to the framework defectivity that occurs in **JUK-14** after contact with liquid water, we carried out soaking for 2, 6, 24, and 48 h to see how it proceeds over time. The experiments showed that as the contact between the **JUK-14** framework and water is increased, its defectiveness rises—from 11% linker loss after 2 h to 18% loss after 48 h (ESI, Figure S7, Table S3). The NMR spectrum taken for **JUK-14_H_2_O** after 24 h of soaking in water shows that a stoichiometric amount of dimethylammonium cations is removed in parallel with the linker removal (ESI, Figure S5). Likewise, this modification was performed in 1 M hydrochloric acid, however, in this case, no correlation is observed between the soaking time and the number of introduced defects. It demonstrates that soaking the **JUK-14** in an HCl solution is a more severe modification, and the relatively short time of framework immersion (2 h) results in reaching a threshold degree of defectiveness of about 30% linker loss (ESI, Figure S8). Elemental analysis and NMR spectrum confirm the complete removal of the Me_2_NH_2_^+^ from the framework (ESI, Figure S5, Table S4). Thus, the TG results, combined with elemental analyses, NMR spectra, and experiments carried out to determine the acidity of the materials (Figure 2c and ESI, Figures S13–S16), allowed us to establish chemical formulas for the materials studied:
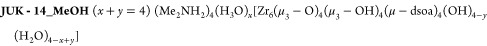

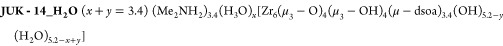

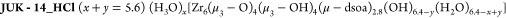


In the aforementioned formulas, for the **JUK-14_MeOH** material, the presence of 4 dimethylammonium cations was assumed because in such a form the material is subjected to most further measurements, whereas for the **JUK-14_HCl** framework, an approximate amount of linker loss equal to 30% was assumed.

**Figure 3:**
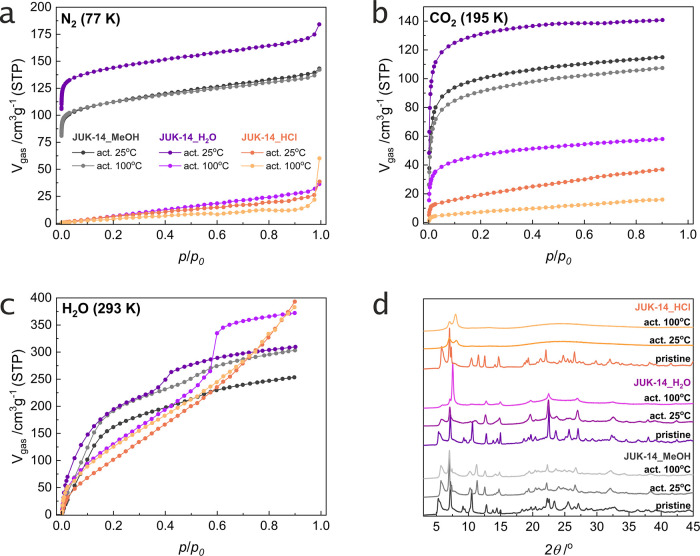
Porosity and stability of **JUK-14** derivatives: physisorption isotherms of (a) N_2_ at 77 K, (b) CO_2_ at 195 K, and (c) H_2_O at 293 K after activation at 25 or 100 °C. Curves represent adsorption branches. Desorption branches have been omitted for clarity. Color codes from Figure 3a refer to the (a)–(c) plots. (d) Experimental PXRD patterns of **JUK-14** derivatives as pristine materials and samples after activation under vacuum at 25 or 100 °C (ex situ measurements).

Surprisingly, the framework decomposition temperature is the highest for **JUK-14_HCl** (ESI, Figure S12) and may be related to the absence of dimethylammonium ions, which are present in **JUK-14_MeOH** and **JUK-14_H_2_O** and release dimethylamine at lower temperatures, leading to the decomposition of these MOFs. Thus, on one hand, dimethylammonium ions stabilize stacking by forming inter-layer hydrogen bonds, which facilitates the maintenance of crystallinity over a wider range of temperatures (Figure 3d), but on the other hand, they contribute to the lowering of decomposition temperature. Comparing the two materials containing dimethylammonium ions, however, defects present in **JUK-14_H_2_O** result in its lower thermal stability than **JUK-14_MeOH**.

#### Potentiometric Titration

In order to determine the acidity of the materials, we first attempted to estimate the acidity of the sulfonic groups. In this aim, UV–Vis measurements were carried out for the sulfonated H_2_dsoa-2H linker precursor in aqueous HCl solutions of various concentrations (from 0.1 to 12 M) to determine the approximate pH value at which these groups are protonated/deprotonated (ESI, Figure S13). The absorbance maximum at 208 nm, corresponding to intraligand π–π* transitions, shifts slightly in the pH range from 0.1 to 5 M HCl solutions, and more significantly at 12 M HCl, which indicates that p*K*_a_ of the sulfonic group is below 0. This observation is also consistent with the previous literature report,^15^ in which p*K*_a_ of the sulfonic group in the UiO-66-SO_3_H framework was determined by DFT calculations as −2.3, and indicates that these groups are present in the **JUK-14** material mainly as deprotonated sulfonate groups.

Subsequently, several potentiometric alkalimetric titrations were performed for **JUK-14_MeOH**, **JUK-14_H_2_O**, and **JUK-14_HCl**, to determine the acidity of protons present on zirconium clusters (Figure 2c; the detailed description of the experiment is given in the ESI).^22^ Before each titration, initial pH values of the suspensions have been measured as 5.12, 4.55, and 3.61 for **JUK-14_MeOH**, **JUK-14_H_2_O**, and **JUK-14_HCl**, respectively (ESI, Table S5), which tentatively indicates the increasing acidity of the materials in this order, which is related to the decreasing amount of Me_2_NH_2_^+^. The first derivative of the titration curve for both the **JUK-14_H_2_O** and **JUK-14_HCl** materials shows three explicit maxima, but in addition, other less separated maxima are present (ESI, Figures S15, S16). The three distinct maxima can be assigned to three different types of acidic protons present in these MOFs, i.e., in the μ_3_-OH bridging ligand and in the −OH_2_ and −OH terminal ligands, similarly as it was observed for other hexazirconium-based MOFs.^23^ The occurrence of the other less pronounced maxima may be related to other protons present in the H_3_O^+^ form. In contrast, on the first derivative curve of **JUK-14_MeOH** (ESI, Figure S14), multiple equilibrium points at the initial phase of titration can be identified, even though this material should also have only three main types of acidic protons in the studied pH range (∼3–11). A plausible explanation for this result is that the sample when dispersed in an aqueous solution and then acidified, undergoes dynamic changes, consisting of gradual loss of linkers, which results in the formation of new acidic sites during titration. The p*K*_a_ values for all MOFs were determined as the pH values at one-half of the volume of titrant added to reach the equivalence point (Figure 2c). The values corresponding to the terminal ligands −OH_2_ and −OH (the amount of which differs in the samples) show an increase in the acidity of the materials in the order of **JUK-14_MeOH** < **JUK-14_H_2_O** < **JUK-14_HCl**, which also correlates with an increase in defectivity.

#### Pyridine Adsorption

The differences between the materials were further confirmed by pyridine adsorption monitored by in situ IR measurements (ESI, Figures S18, S19). The whole **JUK-14** family, regardless of defects, have potential Lewis acid sites (LAS) which are CUS at the zirconium clusters after the removal of terminal water molecules and whole linkers in case of defects, as well as Brönsted acid sites (BAS) predominantly in the form of hydroxo and water ligands located on metallic clusters. Based on the appearance of PyH^+^ IR bands, an importantly lower amount of accessible BAS was detected for **JUK-14_MeOH** and **JUK-14_H_2_O**, in contrast to **JUK-14_HCl** (ESI, Figure S18) with the strongest IR signal of protonated pyridine. In these materials, the negative charge of SO_3_^–^ groups is mostly compensated by dimethylammonium cations, which can block the access for the pyridine probe, whereas **JUK-14_HCl** contains no Me_2_NH_2_^+^ ions. On the other hand, the analysis of LAS indicates their highest amount for **JUK-14_HCl**, a slightly lower for **JUK-14_H_2_O** and the lowest for **JUK-14_MeOH**. Qualitatively, these results are consistent with the previous studies (TG-based estimation of the number of missing linkers), since the more defective the material is, the more CUS it has. The amount of LAS in **JUK-14_MeOH** is more than three times lower as compared to the other two variants (**JUK-14_H_2_O** and **JUK-14_HCl**), indicating the largest steric hindrance to pyridine penetration arising from the presence of all linkers. An indicator of the varying strength of LAS is the position of the Py band associated with the Lewis centers. In **JUK-14_HCl**, the high-frequency component increases significantly at 1456 cm^–1^, documenting the appearance of a LAS of the highest strength that is not found in the other two materials. This result is consistent with the material acidity determined from the titration curves (Figure 2c).

### Sorption Properties and Stability

In the previous work, **JUK-14** (after soaking in methanol, here denoted as **JUK-14_MeOH**) was presented as a material that possesses permanent porosity.^21^ Here, we demonstrate an improved analysis of both the porosity and stability of this material (with a slightly modified activation procedure, see ESI), as well as the comparison with **JUK-14_H_2_O** and **JUK-14_HCl** materials.

#### N_2_ Adsorption

Nitrogen sorption measurements (at 77 K) revealed that only the materials soaked in methanol or water show permanent porosity (Figure 3a and ESI, Figure S20), with BET surface areas of 430 and 510 m^2^/g, respectively (calculated for the materials activated at 25 °C). **JUK-14_H_2_O** achieves a 25% higher adsorption capacity than **JUK-14_MeOH** due to the absence of some linkers, which also results in an increase in specific surface area. Nevertheless, while the activation temperature (either 25 or 100 °C) does not influence the sorption capacity of **JUK-14_MeOH** for nitrogen, the activation of **JUK-14_H_2_O** at a higher temperature (100 °C) leads to a loss of its porosity, which is caused by a decrease in the thermal stability of the material due to the occurrence of missing-linker defects, a phenomenon known in the literature.^15,24^ In contrast, **JUK-14** soaked in HCl, regardless of the activation temperature does not adsorb nitrogen. Moreover, regardless of the use of alternative activation methods such as cyclohexane freeze-drying (ESI, Figure S24), the material lacks accessible porosity. These observations indicate the stabilizing role of dimethylammonium cations for the layered **JUK-14** system.

#### CO_2_ Adsorption

Similar adsorption behavior of the studied materials was observed for carbon dioxide at 195 K (Figure 3b and ESI, Figure S21). The most significant difference is observed for **JUK-14_MeOH**, where activation at 25 °C increases the sorption capacity of CO_2_ by about 10% (relative to the material activated at 100 °C). This effect is caused by the incomplete removal of water molecules coordinated with zirconium clusters (after activation at 25 °C), which can interact with CO_2_ on a dipole-quadrupole basis, increasing the sorption capacity. This effect does not occur in the case of nitrogen sorption, since the N_2_ molecule has a negligible electric quadrupole moment. In the case of **JUK-14_H_2_O** and **JUK-14_HCl** materials, the overall results are very similar to those observed for nitrogen adsorption, with the higher sorption capacity toward carbon dioxide than nitrogen attributed to the smaller molecular size of CO_2_ (3.30 vs 3.64 Å), which thus more easily penetrates the partially destroyed framework.

#### H_2_O Adsorption

For water vapor adsorption measurements at 293 K (Figure 3c and ESI, Figure S22), which is important in terms of proton conduction studies, the situation is different because all three materials, regardless of the activation temperature, have significant sorption capacities. The general explanation for these results is both the small size of the water molecule (2.60 Å) and the hydrophilic nature of the framework with which the water molecule can interact. The strong framework hydrophilicity is also confirmed by the large hysteresis loop between adsorption–desorption curves (ESI, Figure S22). When activated at 25 °C, both **JUK-14_MeOH** and **JUK-14_H_2_O** adsorb less water than when activated at 100 °C, because coordinated water molecules are not completely removed at 25 °C, which reduces the sorption capacity of these materials. An interesting result is observed for the **JUK-14_H_2_O** material, where the activation temperature affects not only the sorption capacity but also the shape of the curve. The adsorption curve of the material activated at 25 °C has the characteristic shape of **JUK-14_MeOH**, while the adsorption curve of the material activated at 100 °C has the typical shape of **JUK-14_HCl**. This result is consistent with the PXRD patterns obtained after activation at the given temperature (Figure 3d). The material after soaking in water, activated at 25 °C, retains its crystallinity (similarly to the material after soaking in methanol), so it adsorbs both N_2_ and CO_2_, while the material activated at 100 °C undergoes structural changes and significantly loses its crystallinity and adsorptive capacity towards N_2_ and CO_2_ (similarly to the material after soaking in HCl). The distinctive steps in the water vapor adsorption curves of the **JUK-14_MeOH** and **JUK-14_H_2_O** materials can be attributed to the disruption of hydrogen bonds between the dimethylammonium cation and the framework layers, which, when separated, cause an increase in the sorption capacity of the framework. In the case of **JUK-14_HCl**, the activation temperature does not influence its sorption capacity or the shape of the curve. This is also consistent with the measured PXRD patterns, where no differences are observed between the material activated at 25 and 100 °C. These results indicate that **JUK-14** soaked in HCl loses its long-range ordering and porosity during activation but relatively small H_2_O molecules strongly interacting with the framework can still be adsorbed (as for **JUK-14_H_2_O** activated at 100 °C). The retention of framework integrity, even despite the loss of long-range ordering, is confirmed by IR spectra that remain unchanged regardless of activation temperature (ESI, Figure S23).

In summary, the most thermally stable material is **JUK-14_MeOH**, slightly less **JUK-14_H_2_O**, and the least stable is **JUK-14_HCl**. In the PXRD patterns of the materials that were either soaked in water and activated at 100 °C, or soaked in HCl and activated at 25 or 100 °C, a shift of reflections toward higher 2θ values is observed indicating structural changes (for **JUK-14_H_2_O**), or disappearance of most reflections indicating significant amorphization of **JUK-14_HCl** (Figure 3d). The introduction of missing linker defects into MOFs on one hand can increase their pore accessibility (if appropriate activation methods are used), but on the other hand, reduces their thermal stability. In addition, the presence of an interlayer stabilizer, dimethylammonium cation, greatly enhances the 2D framework robustness by interlayer hydrogen bond formation, creating 3D networks.

### Proton Conduction

#### Humidity-Dependent Conduction

**Figure 4:**
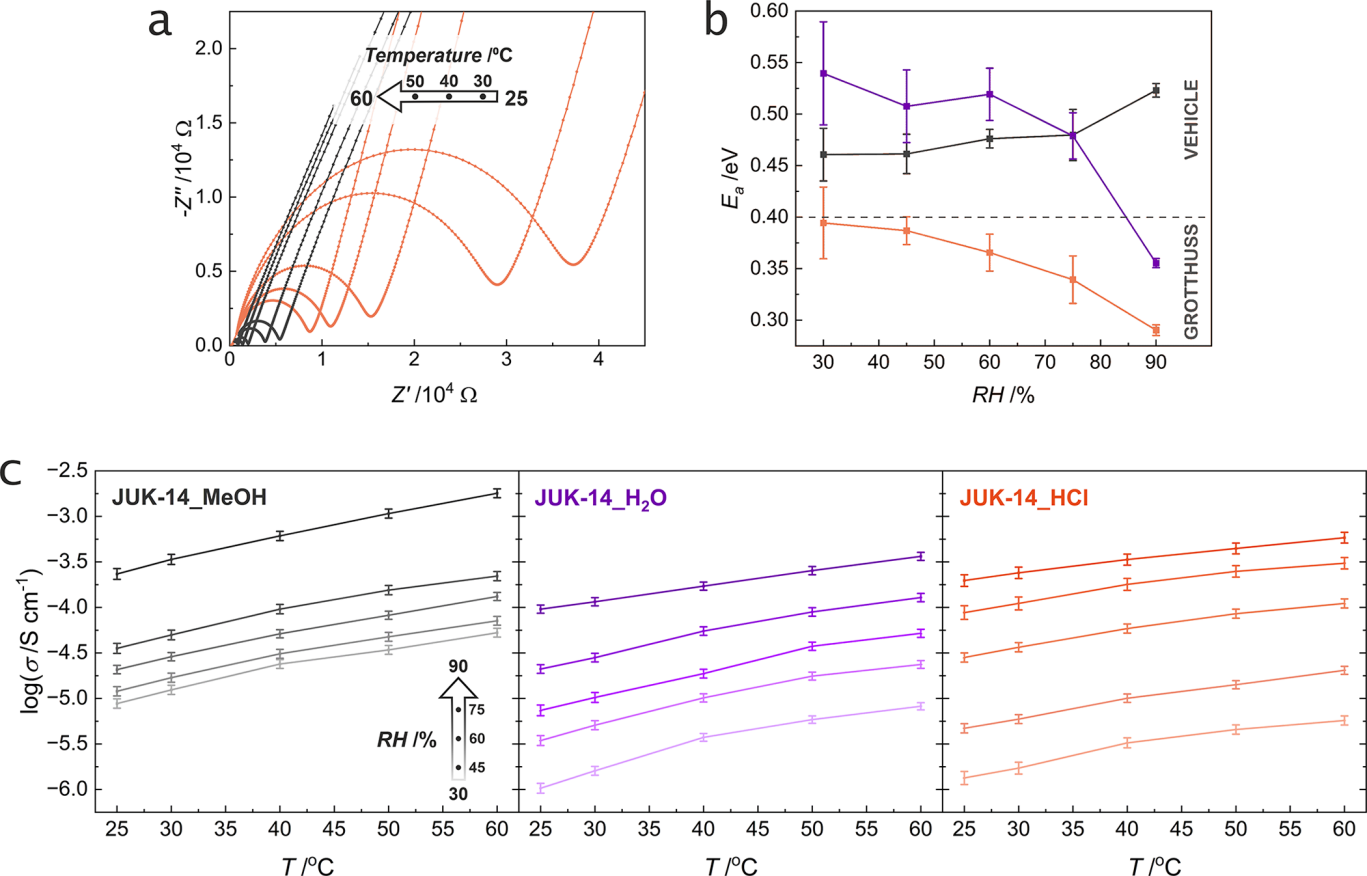
Proton conduction for **JUK-14** derivatives: (a) Nyquist plots (5 MHz–4 Hz) for **JUK-14_MeOH** (gray) and **JUK-14_HCl** (orange) at 30% RH in the 25–60 °C range. (b) Dependence of activation energies (*E*_a_) on relative humidity (RH) for **JUK-14_MeOH** (gray), **JUK-14_H_2_O** (violet), and **JUK-14_HCl** (orange). (c) Dependence of proton conductivity on temperature for various RH values (in the 30–90% RH range) for **JUK-14** derivatives. Error bars are shown for activation energies and conductivities. Values for **JUK-14_H_2_O** were taken from ref (21).

The electrochemical impedance spectroscopy (EIS) measurements were performed for **JUK-14_MeOH** and **JUK-14_HCl** materials at 30, 45, 60, 75, and 90% relative humidity (RH) in the temperature range of 25–60 °C (Figure 4a and ESI, Figures S25, S26). In order to get a complete picture of the effect of defects and the presence of the Me_2_NH_2_^+^ ions on proton conductivity, the results obtained were also compared with the values reported earlier for the **JUK-14** MOF after soaking in water, which is denoted in this work as **JUK-14_H_2_O**.^21^ Comparing **JUK-14_MeOH** and **JUK-14_H_2_O**, higher conductivity is observed for the methanol-soaked material (Figure 4c), which, like the water-soaked one, has dimethylammonium cations, but is not defective, i.e., contains more sulfonate groups, which leads to the conclusion that the poorly basic, but hydrophilic sulfonate groups may contribute to the proton conductivity by forming a more dense hydrogen bond network. Comparing **JUK-14_H_2_O** and **JUK-14_HCl**, higher conductivity is achieved by the hydrochloric acid-soaked material, which has twice as many defects as the water-soaked material, but no dimethylammonium cations. These results show that replacing Me_2_NH_2_^+^ cations with a proton is a key factor in increasing conductivity. **JUK-14_MeOH** exhibits a higher conductivity than **JUK-14_HCl** at 298 K and up to 45% RH. At higher humidities of 60 and 75% RH, this trend is reversed with **JUK-14_HCl** outperforming **JUK-14_MeOH**. At 90% RH, **JUK-14_MeOH** is again more conductive than **JUK-14_HCl** (Figure 4c and ESI, Figures S27, S28). The 60–75% RH range is the region in which the water adsorption isotherms intersect (Figure 3c) and higher water sorption capacities tend to be reached by **JUK-14_HCl**, resulting in a significant enhancement of its conductivity. However, despite the higher water sorption capacity of **JUK-14_HCl** achieved at 90% RH, its conductivity is below **JUK-14_MeOH**. This fact might be related to the actual mechanism of proton conduction in the latter system in which both Me_2_NH_2_^+^ and protonated water molecules facilitate proton mobility. The superiority of **JUK-14_MeOH** conductivity is more pronounced at higher temperatures (ESI, Table S6). Considering most optimal studied conditions, i.e., 90% RH and 60 °C, the highest conductivity is exhibited by **JUK-14_MeOH**, slightly lower by **JUK-14_HCl**, and the lowest by **JUK-14_H_2_O**, with corresponding values of 1.8 × 10^–3^, 5.8 × 10^–4^, and 3.6 × 10^–4^ S cm^–1^. In order to provide more insight into the calculated values, we performed uncertainty calculations for the conductivity measurements. The results showed that the percentage mean uncertainty value for each material was: for **JUK-14_MeOH** – 12.2%, **JUK-14_H_2_O** – 11.4%, and **JUK-14_HCl** – 14.0% (Figure 4c). It can, therefore, be concluded that the impedance measurements exhibit high accuracy. The results suggest that an optimal proton conductor **JUK-14** should have no Me_2_NH_2_^+^ cations compensating the charge of sulfonate groups, and no missing-linker defects. In the literature, there is only one example of the effect of replacing a dimethylammonium cation with an ammonium cation.^25^ This substitution increases the conductivity by more than two orders of magnitude, which is consistent with our observations that smaller and more mobile cations are able to transport the charge more efficiently. In our case, the location of protons that compensate the charge of sulfonate groups on zirconium clusters (e.g., protonating water molecules or hydroxo groups) and not on dimethylammonium cations leads to higher proton conductivities.

In general, activation energies for proton conduction vary with humidity, i.e., increase slightly for the material without defects and decrease significantly for defective materials (Figure 4b and ESI, Figure S27). This is associated with proton mobility, which especially, in materials with reduced density, i.e., soaked in water or in acid, increases significantly upon saturation with water that leads to the increase of density of conduction paths in defective materials. This is further supported by water vapor adsorption isotherms, which are much steeper for defective materials. Given that some of the calculated values are on the borderline between the two mechanisms, we conducted an uncertainty analysis of determination of *E*_a_ values. This study showed that the percentage uncertainty of the *E*_a_ calculations ranges from 1.2 to 9.3% (depending on the accuracy of a fit to the Arrhenius linear plot; ESI, Figure S23), with an average of 4.7%. This result, particularly for **JUK-14_HCl** measured at lower humidities, indicates that conduction can occur by a mixed mechanism, however, at humidities of 60% and higher it indicates a predominant contribution by the Grotthuss mechanism. Both **JUK-14_H_2_O** and **JUK-14_MeOH** have higher activation energies (mostly in the 0.45–0.55 eV range), which suggest the participation of the dimethylammonium or hydronium cations in the conduction pathway and a vehicle mechanism of proton transport.

#### Anhydrous Conduction

For **JUK-14_MeOH**, impedance measurements were also carried out under anhydrous conditions (100–160 °C, ESI, Figures S29, S30, Table S7). The PXRD patterns confirm the stability of MOFs during these measurements, similarly as during impedance measurements under controlled humidity (ESI, Figures S6, S31). Equivalent circuit modeling performed for selected Nyquist plots indicates the presence of resistive components originating from: bulk, grain boundary, and electrode/electrolyte interface (ESI, Figures S32, S33). The maximum conductivity value (at 160 °C) reached 3.7 × 10^–7^ S cm^–1^ for **JUK-14_MeOH**, which exceeded the values obtained for the water-soaked material by approximately two orders of magnitude. A similar correlation is observed in the measurements with controlled humidity, however, in this case the conductivity of **JUK-14_MeOH** is only ca. five times higher than **JUK-14_H_2_O**. A plausible explanation for this observation is lower thermal stability of the **JUK-14_H_2_O** material, which becomes amorphous at high temperatures.^21^ In addition, under humid conditions, defective sites on the zirconium cluster can be saturated by terminal water molecules, which, with their weakly acidic properties (Figure 2c), can participate in proton transport. On the other hand, the acidity of the material determined by titration indicates that **JUK-14_MeOH** has the lowest acidity (highest p*K*_a_). However, these p*K*_a_ values concern only the protons on zirconium clusters and not sulfonic groups, which, in the case of **JUK-14_HCl** may be partially protonated (ESI, Figure S13). Consequently, it can be concluded that MOFs ability to conduct protons is a complex relationship between the acidity of a material, its ability to adsorb water, and its thermal stability.

## Conclusions

In summary, we have introduced defects into a 2D zirconium-based metal–organic framework using post-synthetic modifications to gain insight into the performance-stability correlations of proton conductive MOFs. All studied 2D **JUK-14** MOFs are based on linkers with hydrophilic sulfonate groups, which are weak Bronsted bases. In the “ideal” MOF (**JUK-14_MeOH**), each hexa-zirconium cluster contains eight sulfonate groups, which are compensated by eight dimethylammonium cations; however, during activation, half of these cations are removed from the framework. Increasing material defectivity with (partially or fully) removed dimethylammonium cations results in an enhancement of the framework acidity (in the order **JUK-14_MeOH**, **JUK-14_H_2_O**, and **JUK-14_HCl**), as confirmed by potentiometric titration experiments and pyridine adsorption. Nevertheless, these modifications also have a negative effect on the stability of the analyzed MOFs, which manifests as amorphization and a lack or reduced adsorption capacity toward N_2_ and CO_2_ when harsh activation methods (which, however, do not have a destructive impact on “ideal” material) are used. The “ideal” material shows moderate to high proton conductivities and the charge transport occurs according to the vehicle mechanism regardless of humidity (in the 30–90% RH range). The introduction of missing-linker defects (30%) combined with a complete removal of dimethylammonium ions leads to a significant increase of the MOF acidity (in **JUK-14_HCl**) and a change of predominant transport mechanism for Grotthuss proton hopping. The change of mechanism can be explained by the increase of network acidity which facilitates proton mobility when voltage is applied. As a result, the proton conductivity of this defective MOF is even higher than that of the “ideal” MOF at 60 and 75% RH, which indicates the formation of an optimal hydrogen network for proton hopping under these conditions. For comparison, the less-defective **JUK-14_H_2_O**, i.e., with a smaller number of missing linkers (15%) and partially removed Me_2_NH_2_^+^ ions, exhibits the lowest conductivities and crossover of mechanisms, dependent on conditions. In general, all modifications have a two-fold impact: the “mild” defectivity (in **JUK-14_H_2_O**) can improve adsorption capacity but at the expense of reduced stability. Similarly, the presence of interlayer entities (here dimethylammonium cations) can decrease proton conductivity but, on the other hand, makes the material much more robust.

Overall, this work provides an insight into the underdeveloped defect-property relationship for two-dimensional MOFs. These layered MOFs, in contrast to their three-dimensional counterparts, have nodes with reduced connectivity and, consequently, with coordinatively unsaturated sites, already in their defect-free forms. While such unsaturated sites may reduce material stability, they are often favorable in catalysis or may enhance proton conductivity by the presence of terminal acidic ligands. Our study shows the intricate relationship between the performance, stability, and defects for the first time in a two-dimensional proton conductive MOF. These findings emphasize the role of missing sulfonate linkers and associated counterions in a layered MOF and may serve as a guidance for designing efficient and stable MOF-based proton conductors in the future. They also highlight the underestimated contribution of zirconium cluster acidity toward proton conduction, as well as confirm the fact that pendant sulfonic groups of linkers occur in the deprotonated SO_3_^–^ form and not as SO_3_H, often presented in the literature.

## Supporting Information Available

Defective metal-organic framework investigated for proton conductivity; details of synthetic procedures, sample handling, physical measurements and additional IR, PXRD, EIS, and adsorption data; Characterization of JUK-14 derivatives; sorption properties of JUK-14 derivatives; proton conduction properties of JUK-14 derivatives (PDF)
