# Supplementary material for: Effect of Missing-Linker Defects and Ion Exchange on Stability and Proton Conduction of a Sulfonated Layered Zr-MOF
Source: ACS Appl Mater Interfaces. 2023 Jun 2;15(23):28184–92. doi: 10.1021/acsami.3c03873 (PMC10273224; doi:10.1021/acsami.3c03873)
Supplement: Supplementary file 1 — am3c03873_si_001.pdf [file am3c03873_si_001.pdf]

Supporting Information

Effect of missing-linker defects and ion exchange on stability and proton conduction of a sulfonated layered Zr-MOF

Monika Szufla,[a,b] Jorge A. R. Navarro,[c] Kinga Góra-Marek,[a] and Dariusz Matoga*[a]

[a]Faculty of Chemistry, Jagiellonian University, Gronostajowa 2, 30-387 Kraków, Poland

[b] Doctoral School of Exact and Natural Sciences, Jagiellonian University in Kraków, Gronostajowa 2, 30-387 Kraków, Poland

[c] Departamento de Química Inorgánica, Universidad de Granada, 18071 Granada, Spain

*Corresponding author: dariusz.matoga@uj.edu.pl

Table of Contents

Defective MOFs investigated for proton conductivity (literature data) 2

Details of physical measurements 2

Synthetic procedures 4

Figures and Tables 7

A. Characterization of JUK-14 derivatives 7

B. Sorption properties of JUK-14 derivatives 19

C. Proton conduction properties of JUK-14 derivatives 21

References 28

Defective MOFs investigated for proton conductivity (literature data)

Table S1. Defective metal-organic framework investigated for proton conductivity.

Material	Linker	Net dimensionality	Number of linkers per cluster/ Cluster connectivity[a]	Number of defects/ Percentage of defects	Max. proton conductivity[b] σ / S cm^{-1}	Refs.
UiO-66	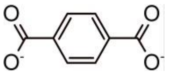	3D	6/12	1.0/17%	6.8×10^{-3} (65 °C, 95% RH)	2015[1]
UiO-66-SO_3H	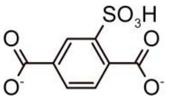	3D	6/12	2.0/33%	5.6×10^{-3} (65 °C, 95% RH)	2015[2]
UiO-66	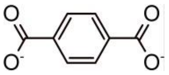	3D	6/12	1.8/30%	3.0×10^{-2} (100 °C, 98% RH)	2022[3]
MOF-808	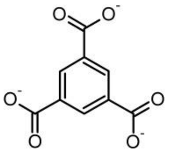	3D	2/6	0.5/25%	2.6×10^{-1} (80 °C, 98% RH)	2022[4]
ZrTST	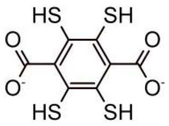	3D	6/12	2.2/37%	1.9×10^{-3} (90°C, 90%RH)	2022[5]
ZrTSaT	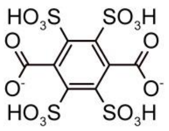			not specified, but not less than 2.2/37% - ZrTST derivative	3.7×10^{-1} (90°C, 90%RH)	

[a] Numbers for ideal, nondefective structures

[b] Maximum reported values for structures with corresponding number of defects (fifth column).

Details of physical measurements

Carbon, hydrogen and nitrogen were determined using an Elementar Vario MICRO Cube elemental analyzer.

Thermogravimetric analyses (TGA) were performed on a Mettler-Toledo TGA/SDTA 851e instrument with a heating rate of 10°C min^{-1} in a temperature range of 25 – 900 °C (approx. sample weight of 10 mg). The measurements were performed at atmospheric pressure under air flow.

FTIR spectra were recorded on a Thermo Scientific Nicolet iS10 FT-IR spectrophotometer equipped with an iD7 diamond ATR attachment.

Powder X-ray diffraction (PXRD) patterns were recorded at room temperature (295 K) on a Rigaku Miniflex 600 diffractometer with Cu-Kα radiation (λ = 1.5418 Å) in a 2θ range from 3° to 45° with a 0.02° step and 3° min^{-1} scan speed. Diffraction patterns for the samples after

titration were obtained on a D2 PHASER Bruker diffractometer using Cu Kα radiation (λ = 1.5418 Å) in 5 - 35° 2θ range with 0.05° steps.

Potentiometric titration was performed using a DMS Titrando 888 automatic titrator (Metrohm) set at the mode to collect the equilibrium pH. The initial samples (~ 50 mg) were ground and dispersed in $NaNO_3$ (50 mL, 0.01 M), equilibrated overnight and continuously saturated with N_2. Before the measurement, samples were acidified with HCl (0.10 M) to pH ~3.0 and then titrated with NaOH (0.10 M) to a pH of 11. The injection rate was 0.01 mL min^{-1}. Equivalence points were obtained as maxima on the first derivative curve of the collected data (the titration curve of pH as a function of volume of titrant added). The pK_a values were determined as the pH in half of the titrant volume added to reach the equivalence point.[6] The derivative curves were smoothed using the Savitzky-Golay method with 60 points of window for JUK-14_MeOH and JUK-14_H_2O and 180 points of window for JUK-14_HCl (OriginPro 2023 software).

UV-Vis spectra were recorded on Shimadzu UV-1800 UV spectrophotometer in the wavelength range 200 – 800 nm with a 0.05 nm step. The measurements were carried out on $1.25 \cdot 10^{-5}$ M solutions of H_2dsoa-2H in various concentrations of aqueous solutions of hydrochloric acid (from 0.001 to 12 M).

^1H NMR spectra were recorded on a Jeol 400 spectrometer. The chemical shifts (δ) are reported in parts per million (ppm) on a scale downfield from tetramethylsilane. The NMR spectra were referenced internally to the residual proton resonance in DMSO-d_6 (δ 2.50 ppm).

Nitrogen (77 K), carbon dioxide (195 K) and water (293 K) adsorption/desorption studies were performed on an Autosorb iQ-C-XR-XR EPDM volumetric analyzer (Quantachrome Instruments). Prior to the physisorption measurements, samples were activated at 25 or 100 °C (specified for a given measurement in manuscript) under vacuum for 1 h. A temperature of 77 K was achieved by a liquid nitrogen bath and 195 K was achieved by dry ice/acetone bath

Electrochemical Impedance Spectroscopy (EIS) measurements were performed using Hioki IM3570 impedance analyzer on pre-conditioned samples (see Samples preparation for EIS Measurement below) of a thickness of 0.017 – 0.032 cm, pressed between two platinum electrodes (8.0 mm diameter with a pressure of 9 MPa) in a PTFE tube of a Fine Instruments PIP-260 probe (Elektronika Jądrowa Kraków, Poland), and kept in KK 115 TOP+ climatic chamber with an ultrasonic humidifier and a temperature control (the measurements with humidity control) or in a vacuum dryer Memmert, VO 200 (the measurement at the temperatures above 100 °C). The measurements were carried out in a quasi-four-probe setup in a frequency range from 4 Hz to 5 MHz, potential of 100 mV, alternating current and temperature range from 25 to 60 °C (for the measurements in 30 – 90% RH) or from 100 to 160 °C (for the anhydrous measurement), and with pre-measurement sample conditioning for 1 h at each temperature. The proton conductivity (σ, S cm^{-1}) of each sample was estimated by using the equation:

$$\sigma = L/(RA)$$

where L (cm) is the sample thickness and A (cm^2) is the cross-sectional area of the measured pellet; R (Ω) is the resistance as the real part of the measured impedance.

IR measurements during the pyridine adsorption were carried out with a Bruker Vertex 70 spectrometer. The MOFs were dispersed on silicon wafer (1 mg×cm^{-2}), which were placed in a quartz cell equipped with the CaF_2 windows. Prior to the experiments, the samples were degassed at 50°C for 60 min under vacuum (10^{-5} bar) and cooled down to room temperature. Then upon admission of 10 Torr of pyridine (Py) the materials were heated gradually to 80°C (with a rate of 1 ^{o}C/ min) and the spectra were collected. The spectra presented in the paper are difference spectra (from the spectrum after Py adsorption, the spectrum of the material before probe adsorption was subtracted). The spectra were normalized to the same mass of the sample.

Synthetic procedures

All chemicals and solvents (of analytical grade) were purchased from commercial sources (Merck, Avantor, Polmos) and were used without further purification. Ethanol (Polmos) contained water 8% by volume.

Synthesis of H_2dsoa-2Cl precursor

4,4'-Oxybis[3-(chlorosulfonyl)benzoic] acid (H_2dsoa-2Cl, Figure S2) was prepared according to the published method.[7] In the round bottom flask, the mixture of 4,4'-oxydibenzoic acid (5.00 g, 0.019 mol) and HSO_3Cl (50.0 ml, 0.760 mol) was heated at 120 ^{o}C under reflux for 4 h. After that, the dark solution was poured on ice to destroy the excess of chlorosulfonic acid. The white precipitate formed was filtered off, washed with cold water (0 ^{o}C) until neutral pH was reached, and dried in a desiccator over $CaCl_2$ overnight. The identity of H_2dsoa-2Cl was verified as in reference.[8]

Synthesis of H_2dsoa-2H precursor

4,4'-Oxybis[3-(sulfonyl)benzoic] acid (H_2dsoa-2H, Figure S2) was prepared was prepared according to the published method.[8] To the saturated Na_2SO_3 solution in H_2O (5.0 mL) H_2dsoa-2Cl (0.32 g, 0.70 mmol) acid was added. Then the 32% NaOH solution was added dropwise to reach pH of ca. 10 and the resulting solution was stirred overnight at RT. After that, the solution was placed in an ice bath and acidified with 25% HCl to reach pH of 1. The product formed was filtered off, washed with cold water and dried at 60 ^{o}C. The identity of H_2dsoa-2H was verified as in reference.[8]

Synthesis of JUK-14

JUK-14 was prepared according to a slightly modified (upscaled) published method.[8] $ZrCl_4$ (274 mg, 1.18 mmol), H_2dsoa-2Cl (683 mg, 1.50 mmol) were dissolved in DMF (25.0 mL) and CH_3COOH (15.5 mL, 320 mmol), placed in a 100 ml glass bottle, sonicated (30 s) and heated at 120 °C for 14 days. Colorless crystals of JUK-14 were filtered off, washed with DMF and dried in air at room temperature. The identity of JUK-14 was verified as in reference (IR, PXRD, EA).[8]

Synthesis of JUK-14_MeOH

150 mg of JUK-14 was immersed in 10 mL of methanol. During the first hour of conditioning, the solvent was changed three times (at 15-minute intervals). In the last portion of the liquid the sample was conditioned for the next 23 hours. After that, white powder was filtered on a funnel (sinter G4), washed with 3 ml of methanol and dried in air at ambient conditions.

For impedance and sorption measurements JUK-14_MeOH sample is firstly activated at an elevated temperature and reduced pressure, and then (for impedance measurements) conditioned in various humidity. To investigate the stability of the material in high humidity, the sample was subjected to a series of modifications, reproducing the conditions to which it is exposed prior to impedance measurements. Thus, JUK-14_MeOH was: 1) activated at 100°C, 10 mbar for 2 h, 2) conditioned at 90% RH and 60°C for 16 h, and finally 3) immersed in methanol for 2 h (to remove any possible non-bound linkers). During the conditioning, the solvent was changed three times and after that, the powder was filtered on a funnel (sintered glass funnel G4), washed with 3 ml of methanol and dried in air at ambient conditions.

Synthesis of JUK-14_H_2O

The synthesis was carried out in a manner analogous to that described above, but instead of methanol, distilled water was used. In addition, the kinetics of JUK-14 defecting in water was investigated by performing soaking for 2, 6, 24 and 48 h.

Synthesis of JUK-14_HCl

The synthesis was carried out in a manner analogous to JUK-14_H_2O, but instead of water, 1 M aqueous solution of HCl (pH = 0) was used. Additionally, after filtering, the powder was washed with three portion of distilled water, 3 ml each (to obtain a neutral pH).

After the aforementioned modifications, to investigate the stability and defectivity of the materials JUK-14_MeOH, JUK-14_H_2O and JUK-14_HCl, elemental and thermogravimetric analysis measurements were carried out and powder diffraction patterns and IR spectra were recorded (see *Characterization of JUK-14 derivatives* section).

Sample preparation for EIS measurements

Prior to each series of EIS measurements at a given relative humidity (RH), 20-30 mg of the JUK-14_MeOH, JUK-14_H_2O or JUK-14_HCl was ground in a mortar and equilibrated in a humidity chamber for at least 16 hours at a specified RH (90, 75, 60, 45 or 30%) at 25 ^{o}C. Before equilibration, JUK-14_MeOH sample was additionally activated for 2 h in vacuum dryer (100 ^{o}C, 10 mbar) to remove methanol from the framework pores.

Before the measurements in anhydrous environment, the same amount of sample was equilibrated in a vacuum dryer for at least 16 h at 100 ^{o}C (atmospheric pressure) to completely remove solvents from the framework pores.

Calculating the number of defects

The number of defects was estimated using the thermogravimetric analysis. An exemplary plot of TG analysis was presented in Figure S1.

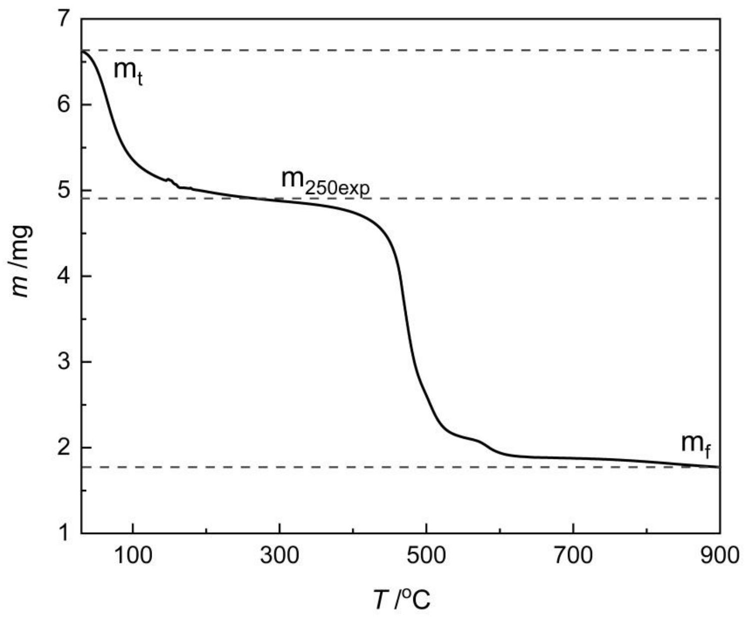

Figure S1. An exemplary plot of thermogravimetric analysis to illustrate the method of calculating defects.

From the TG plot several values were taken, such as:

$\boldsymbol{m_t}$ – total mass of sample (derived from TG in 25°C),

$\boldsymbol{m_{250exp}}$ – experimental mass of sample in 250°C (derived from TG), representing the mass of framework without guest molecules,

$\boldsymbol{m_f}$ – final mass of sample (derived from TG in 900°C), representing the mass of ZrO_2 resulting from framework decomposition.

Additionally, molar mass of the ideal framework was calculated:

$\boldsymbol{M_{MOF}}$ – molar mass of the ideal framework without guest molecules and terminal water ligands

$\boldsymbol{M_{Zr_6\,cluster}}$ – molar mass of $[Zr_6O_4(OH)_4]^{12+}$

for JUK-14_MeOH and JUK-14_H_2O:

$$M_{MOF} = M_{Zr_6\,cluster} + M_{4\cdot OH^-} + M_{4\cdot dsoa-H^{3-}} + M_{4\cdot dma^+}$$

for JUK-14_HCl:

$$M_{MOF} = M_{Zr_6\,cluster} + M_{4\cdot OH^-} + M_{4\cdot dsoa-2H^{2-}}$$

Using these values, the $m_{250theor}$ was calculated with following equation:

$$\boldsymbol{m_{250theor} = \frac{M_{MOF}}{6 \cdot M_{ZrO_2}} \cdot m_f}$$

where:

$\boldsymbol{m_{250theor}}$ – theoretical mass of sample in 250°C, assuming no missing linker defects,

$\boldsymbol{M_{ZrO_2}}$ – molar mass of ZrO_2.

Using these values, the number of linkers was calculated with following equation:

$$n_{linker} = 4 \cdot \frac{m_{250exp} - m_f}{m_{250theor} - m_f}$$

where:

n_{linker} – the real number of linkers in MOF per Zr_6 cluster

Figures and Tables

A. Characterization of JUK-14 derivatives

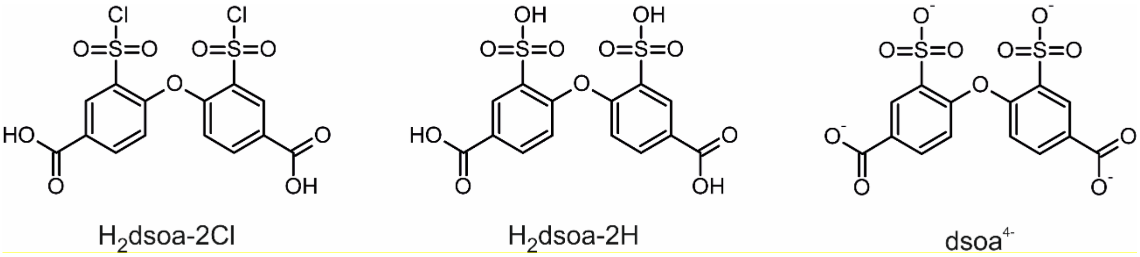

Figure S2. Structural formulas of used linkers precursors: H_2dsoa-2Cl (left) and H_2dsoa-2H (middle) as well as the actual form of the linker in JUK-14 – dsoa^{4-} (right)**.**

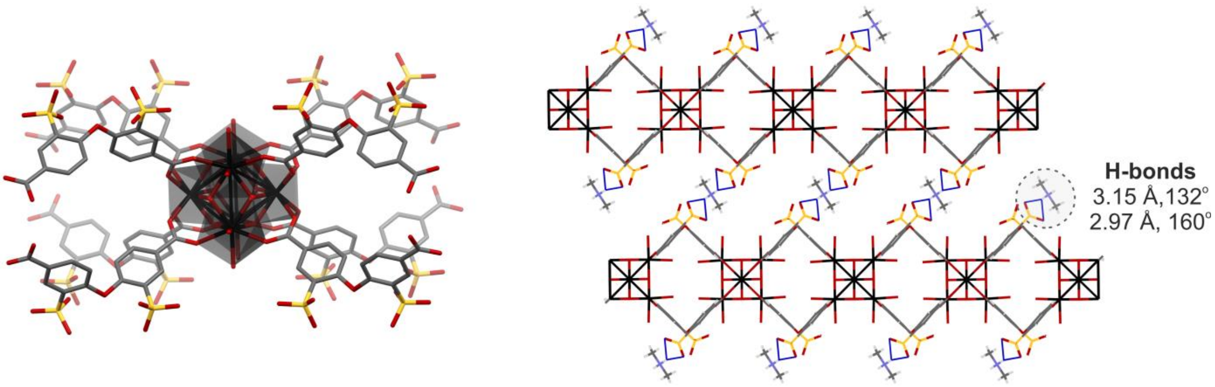

Figure S3. Crystal structure of JUK-14: left: Zr_6 oxocluster with coordinated linkers (hydrogen atoms were omitted for clarity); right: view of the stacked layers along the [010] crystallographic direction with highlighting the hydrogen bonds (blue) between the $Me_2NH_2^+$ and the framework layers. S-yellow, O-red, N-blue, C-grey, H-light grey, Zr-black.

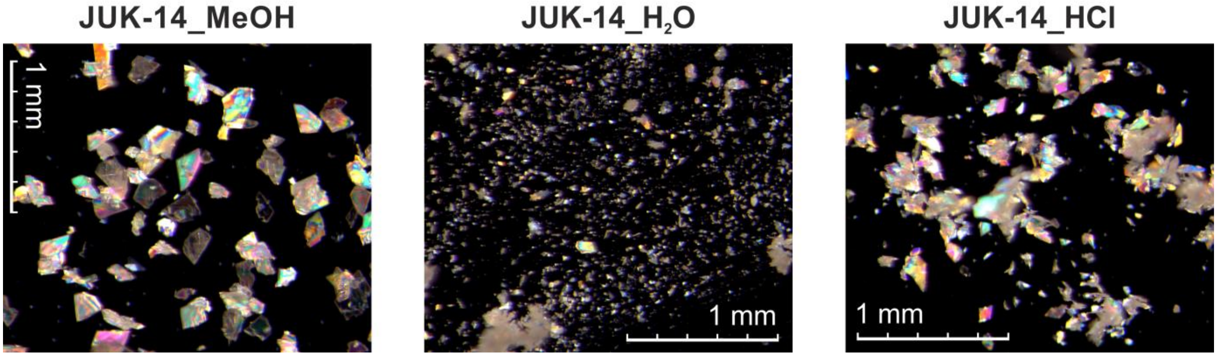

Figure S4. Optical images of JUK-14_MeOH, JUK-14_H_2O and JUK-14_HCl crystals in a polarized light.

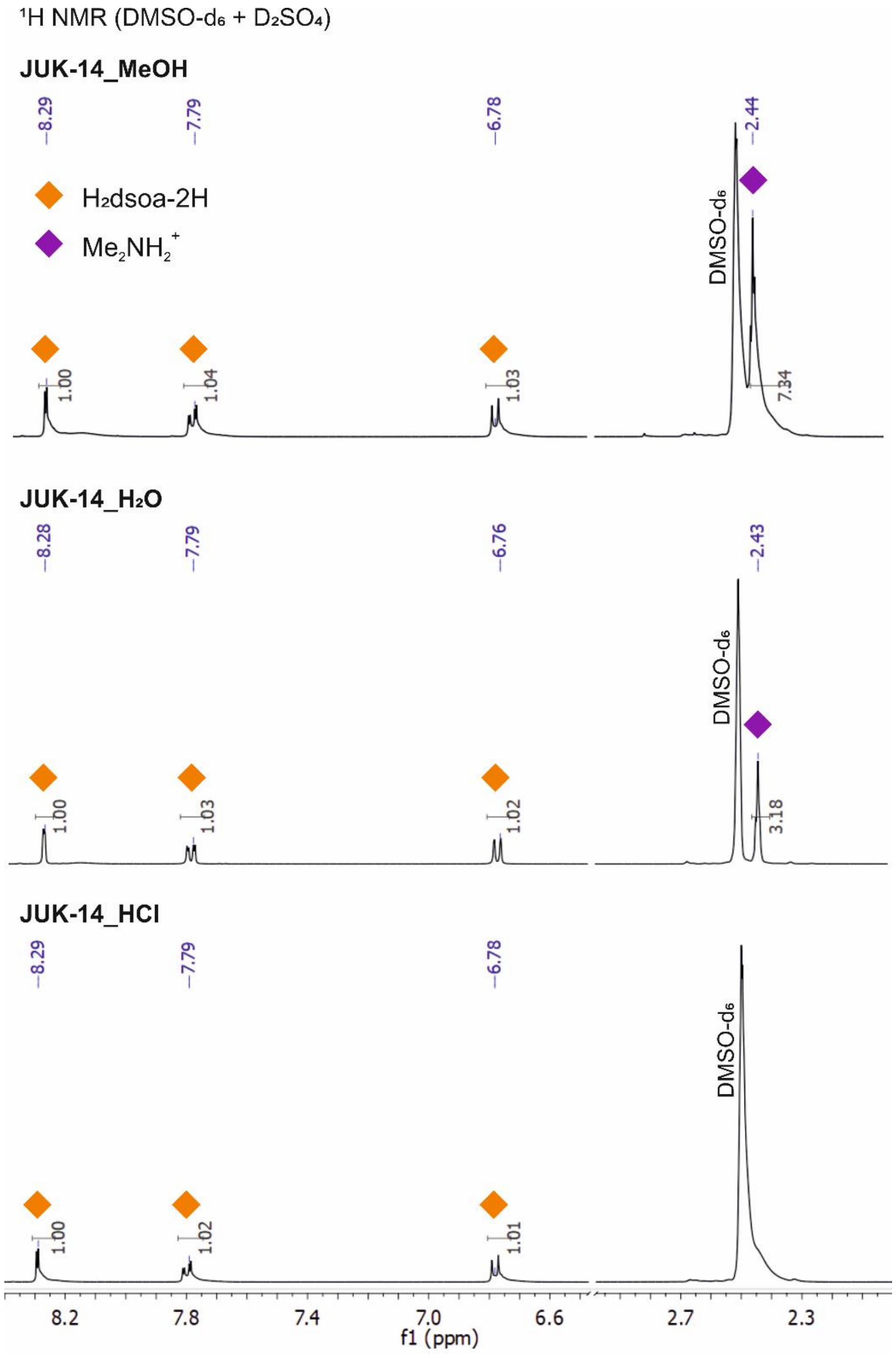

Figure S5. ^{1}H NMR spectra for JUK-14_MeOH, JUK-14_H2O and JUK-14_HCl frameworks digested in D_2SO_4/ DMSO-d_6. The peaks of the dicarboxylic linker are indicated by orange symbols and dimethylammonium cation – violet.

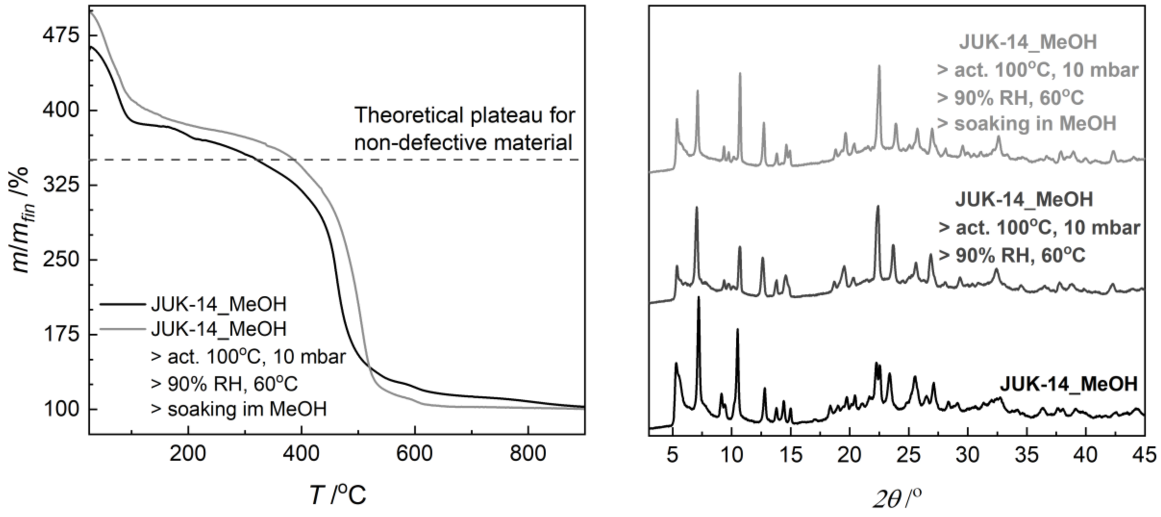

Figure S6. Left - TGA curves (related to ZrO_2) for JUK-14_MeOH (black) JUK-14_MeOH after successive modifications, that is: activation at 100°C and 10 mbar, conditioning at 90% RH and 60°C, and soaking in MeOH (grey). The position of the theoretical TGA plateau, corresponding to the number of linkers equal to 4 (per Zr_6 cluster), is marked by a dashed line. Right – corresponding PXRD patterns of JUK-14_MeOH after specified modifications.

Table S2. Elemental analysis of JUK-14_MeOH and JUK-14_MeOH after activation at 100°C and 10 mbar, conditioning at 90% RH and 60°C, and soaking in MeOH.

		N	C	H	S
**JUK-14_MeOH**	**exp**	2.77	28.03	4.38	8.11
	**calc. for:** $C_{72}H_{128}N_8O_{72}S_8Zr_6$	3.60	27.76	4.33	8.23
**JUK-14_MeOH** > act. 100°C, 10 mbar > 90% RH, 60°C > soaking in MeOH	**exp**	1.72	22.95	4.88	8.09
	**calc. for:** $C_{64}H_{142}N_4O_{95}S_8Zr_6$	1.70	23.35	4.35	7.79

Chemical formulas determined for materials discussed above:

JUK-14_MeOH
$\{(Me_2NH_2)_8[Zr_6(\mu_3\text{-}O)_4(\mu_3\text{-}OH)_4(\mu\text{-dsoa})_4(OH)_4(H_2O)_4]\cdot 15H_2O\}_n$

JUK-14_MeOH after the aforementioned modifications (x + y = 4)
$\{(Me_2NH_2)_4(H_3O)_x[Zr_6(\mu_3\text{-}O)_4(\mu_3\text{-}OH)_4(\mu\text{-dsoa})_4(OH)_{4-y}(H_2O)_{4-x+y}]\cdot 35H_2O\}_n$

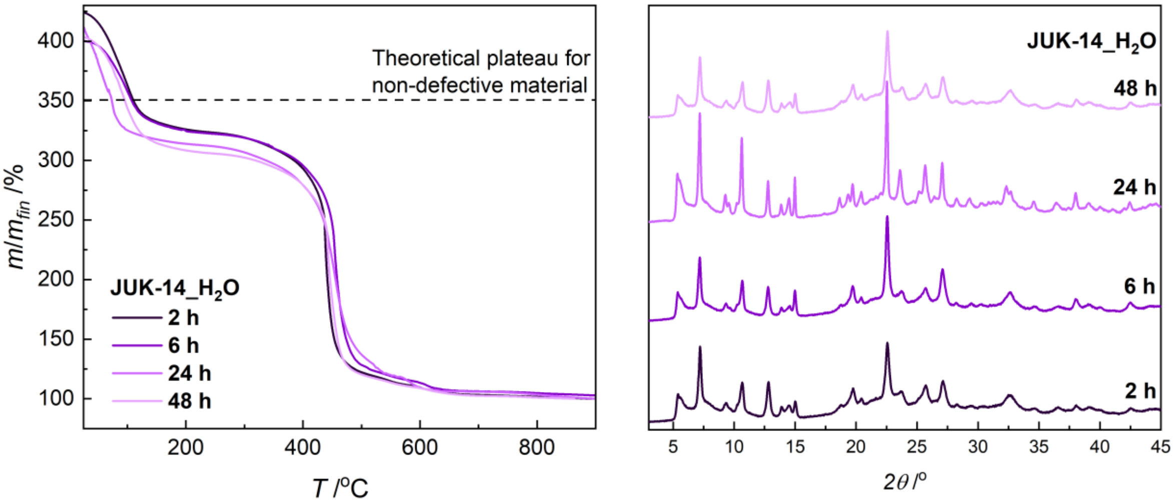

Figure S7. Left - TGA curves (related to ZrO_2) for JUK-14_H_2O soaked in water for a specified time period (2, 6, 24 or 48 h). The position of the theoretical TGA plateau, corresponding to the number of linkers equal to 4 (per Zr_6 cluster), is marked by a dashed line. Calculated real numbers of linkers (per Zr_6 cluster), are included in the Table S3. Right – corresponding PXRD patterns of JUK-14_H_2O .

Table S3. Elemental analysis (left) and calculated from the thermogravimetric analysis (right) defectivity of JUK-14_H_2O soaked in water for a specified time period (2, 6, 24 or 48 h).

**JUK-14_H₂O**		**Elemental analysis**				**Thermogravimetric analysis**	
**time of soaking in H_2O /h**		**N**	**C**	**H**	**S**	**n linker**	**linker loss /%**
**2**	**exp**	1.56	19.92	4.85	8.46	3.56	11
	**calc**	1.64	22.53	4.39	7.53		
**6**	**exp**	1.52	22.23	4.73	8.17	3.54	11
	**calc**	1.64	22.48	4.39	7.51		
**24**	**exp**	1.61	22.13	4.73	6.93	3.38	15
	**calc**	1.60	21.89	4.44	7.31		
**48**	**exp**	1.49	22.32	4.49	8.74	3.28	18
	**calc**	1.67	22.96	4.02	7.67		

Chemical formulas determined for the materials discussed above:

JUK-14_H_2O_2h (x + y = 3.6)

$\{(Me_2NH_2)_{3.6}(H_3O)_x[Zr_6(\mu_3\text{-}O)_4(\mu_3\text{-}OH)_4(\mu\text{-dsoa})_{3.6}(OH)_{4.8-y}(H_2O)_{4.8-x+y}]\cdot 30H_2O\}_n$

JUK-14_H_2O_6h (x + y = 3.5)
$\{(Me_2NH_2)_{3.5}(H_3O)_x[Zr_6(\mu_3\text{-}O)_4(\mu_3\text{-}OH)_4(\mu\text{-dsoa})_{3.5}(OH)_{5.0-y}(H_2O)_{5.0-x+y}]\cdot 30H_2O\}_n$

JUK-14_H_2O_24h (x + y = 3.4)

$\{(Me_2NH_2)_{3.4}(H_3O)_x[Zr_6(\mu_3\text{-}O)_4(\mu_3\text{-}OH)_4(\mu\text{-}dsoa)_{3.4}(OH)_{5.2-y}(H_2O)_{5.2-x+y}]\cdot 30H_2O\}_n$

JUK-14_H₂O_48h (x + y = 3.3)
$\{(Me_2NH_2)_{3.3}(H_3O)_x[Zr_6(\mu_3\text{-}O)_4(\mu_3\text{-}OH)_4(\mu\text{-}dsoa)_{3.3}(OH)_{5.4-y}(H_2O)_{5.4-x+y}]\cdot 20H_2O\}_n$

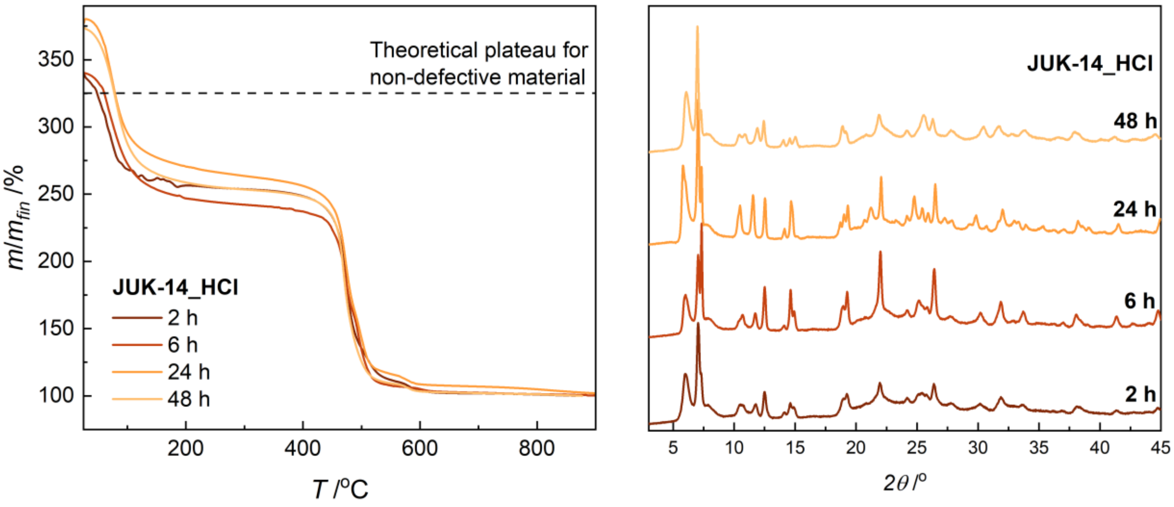

Figure S8. Left - TGA curves (related to ZrO_2) for JUK-14_HCl soaked in water for a specified time period (2, 6, 24 or 48 h). The position of the theoretical TGA plateau, corresponding to the number of linkers equal to 4 (per Zr_6 cluster), is marked by a dashed line. Calculated real numbers of linkers (per Zr_6 cluster), are included in the Table S4. Right – corresponding PXRD patterns of JUK-14_HCl .

Table S4. Elemental analysis (left) and calculated from the thermogravimetric analysis (right) defectivity of JUK-14_HCl soaked in 1 M hydrochloric acid for a specified time period (2, 6, 24 or 48 h).

JUK-14_HCl		Elemental analysis				Thermogravimetric analysis	
**time of soaking in HCl /h**		**N**	**C**	**H**	**S**	**n linker**	**linker loss /%**
**2**	**exp**	0.06	17.31	4.39	7.55	2.74	31
	**calc**	0.00	17.20	4.34	6.57		
**6**	**exp**	0.05	16.72	4.38	6.93	2.55	36
	**calc**	0.00	16.40	4.44	6.26		
**24**	**exp**	0.12	16.13	4.37	6.05	2.86	28
	**calc**	0.00	16.57	4.72	6.33		
**48**	**exp**	0.06	17.16	4.55	7.18	2.75	31
	**calc**	0.00	17.23	4.34	6.58		

Chemical formulas determined for materials discussed above:

JUK-14_HCl_2h (x + y = 5.4)
$\{(H_3O)_x[Zr_6(\mu_3\text{-}O)_4(\mu_3\text{-}OH)_4(\mu\text{-}dsoa)_{2.7}(OH)_{6.6-y}(H_2O)_{6.6-x+y}]\cdot 35H_2O\}_n$

JUK-14_HCl_6h (x + y = 5.2)
$\{(H_3O)_x[Zr_6(\mu_3\text{-}O)_4(\mu_3\text{-}OH)_4(\mu\text{-dsoa})_{2.6}(OH)_{6.8-y}(H_2O)_{6.8-x+y}]\cdot 35H_2O\}_n$

JUK-14_HCl_24h (x + y = 5.8)
$\{(H_3O)_x[Zr_6(\mu_3\text{-}O)_4(\mu_3\text{-}OH)_4(\mu\text{-dsoa})_{2.9}(OH)_{6.2-y}(H_2O)_{6.2-x+y}]\cdot 45H_2O\}_n$

JUK-14_HCl_48h (x + y = 5.6)
$\{(H_3O)_x[Zr_6(\mu_3\text{-}O)_4(\mu_3\text{-}OH)_4(\mu\text{-dsoa})_{2.8}(OH)_{6.4-y}(H_2O)_{6.4-x+y}]\cdot 35H_2O\}_n$

The numbers of defects were estimated according to the method specified in *Calculating the number of defects* section. For the calculation of the theoretical percent composition by mass (N, C, H, S) for JUK-14_H_2O and JUK-14_HCl, an incomplete occupancy of the linkers, in the amount derived from the TG analysis, was assumed. In order to balance the framework charge and fill the coordination sites on zirconium cluster, one linker (2 x COO^-) has been replaced by terminal ligands (2 x H_2O and 2 x OH^-). The proton compensating the negative charge of a sulfonate group is distributed over three possible sites - H_3O^+, OH^- and H_2O.
Further material characterization is presented for the samples soaked for 24 h.

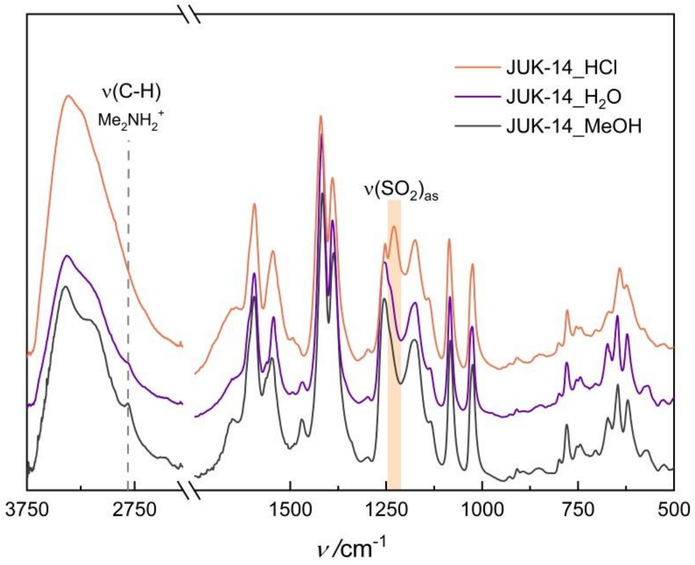

Figure S9. Comparison of IR spectra of JUK-14_MeOH, JUK-14_H_2O and JUK-14_HCl.

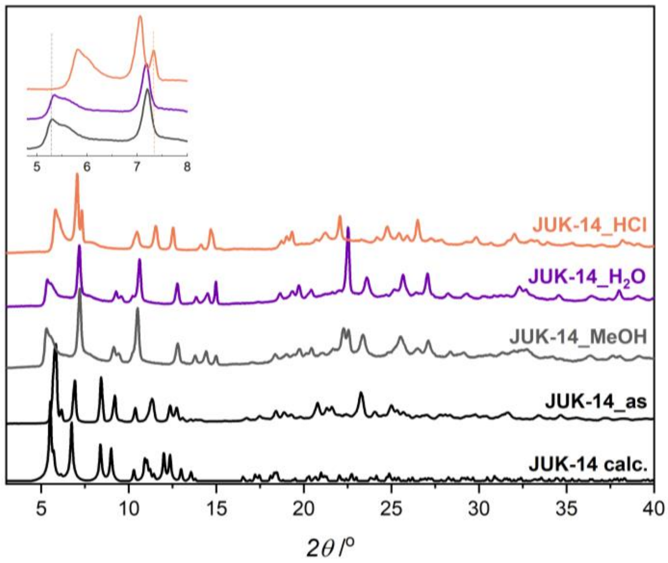

Figure S10. Comparison of PXRD patterns of JUK-14 calculated for the as-synthesized material (JUK-14 calc.), JUK-14_as, JUK-14_MeOH, JUK-14_H_2O and JUK-14_HCl.

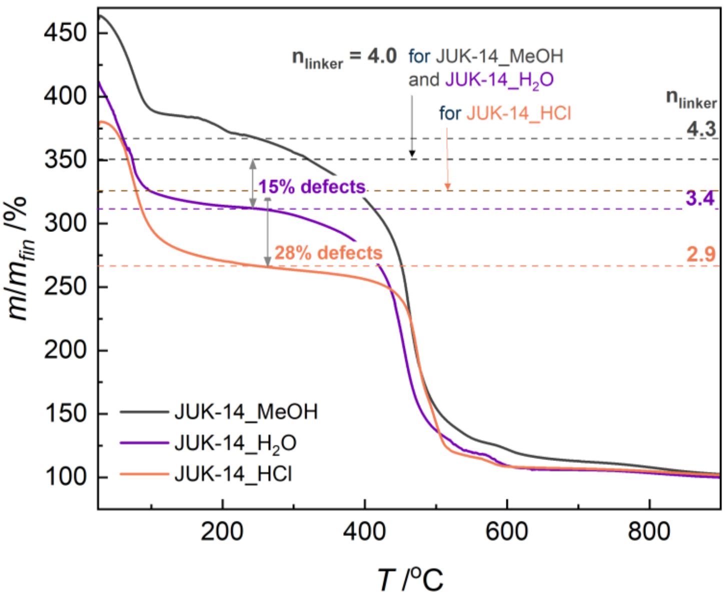

Figure S11. Comparison of TGA curves (related to ZrO_2) for JUK-14_MeOH, JUK-14_H_2O and JUK-14_HCl after 24 h of soaking. The positions of the theoretical TGA plateau, corresponding to the number of linker equal to 4 (per Zr_6 cluster), are marked by dashed lines (indicated by arrows at the top). Real numbers of linkers (per Zr_6 cluster), estimated according to the method specified in *Calculating the number of defects* section, are given on the right side of the graph.

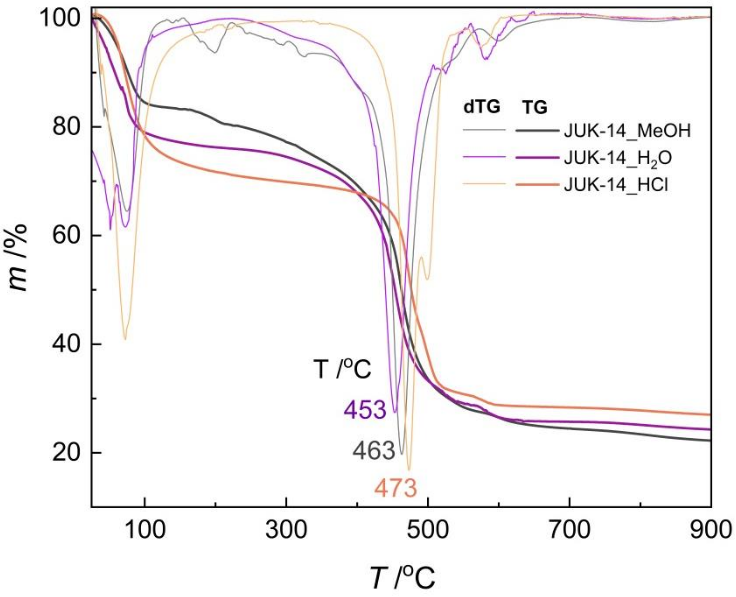

Figure S12. TGA and dTGA curves for JUK-14_MeOH, JUK-14_H_2O and JUK-14_HCl. Temperatures of frameworks decomposition is shown in the color corresponding to the given TG / DTG curve.

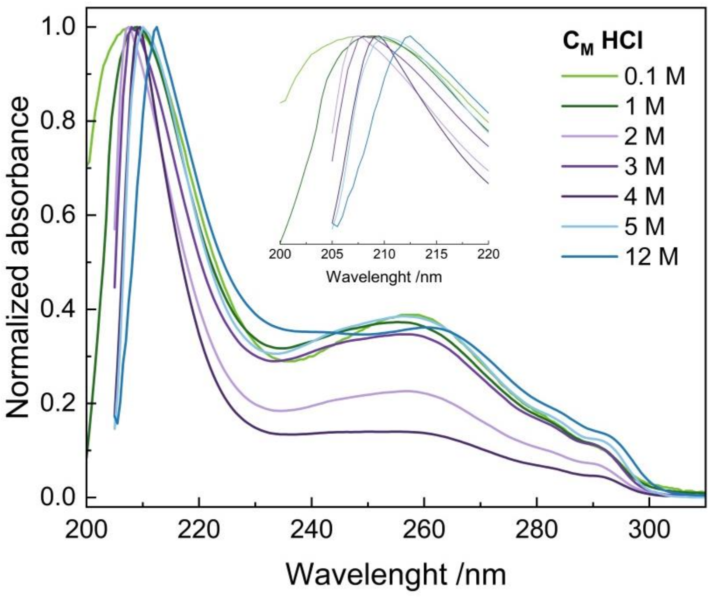

Figure S13. UV-Vis spectra of the pre-hydrolyzed linker precursor (H_2dsoa-2H), in aqueous solutions of hydrochloric acid at concentrations from 0.1 M to 12 M.

Table S5. Comparison of the initial pH values of the MOF suspensions before acidifying.

Material	Initial pH
JUK-14_MeOH	5.12
JUK-14_H_2O	4.55
JUK-14_HCl	3.61

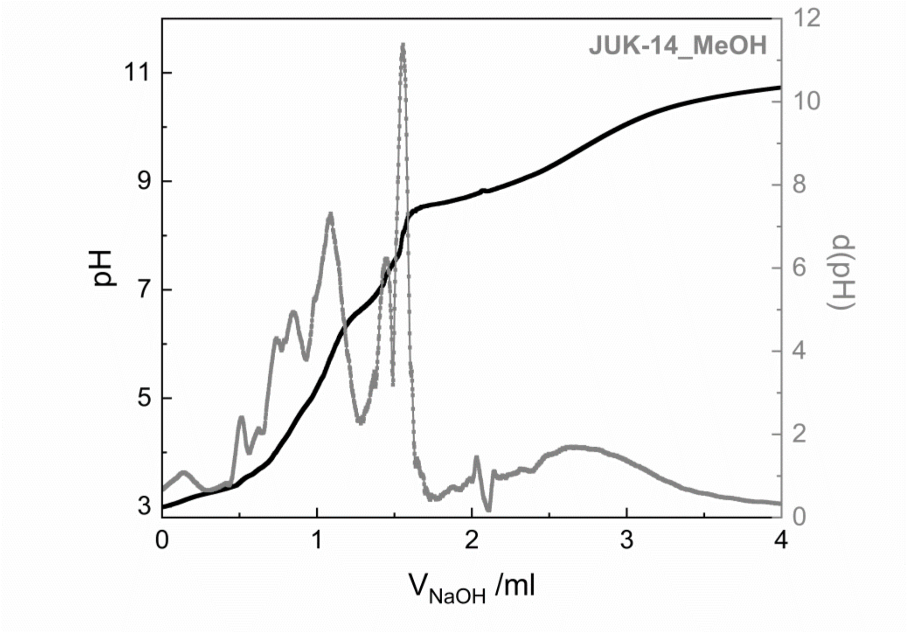

Figure S14. Acid–base titration curve of JUK-14_MeOH (black) and its first derivative curve (gray). To determine the pK_a values of μ$_3$-OH and –OH_2 ligands, the first and second highest intensity maxima were considered.

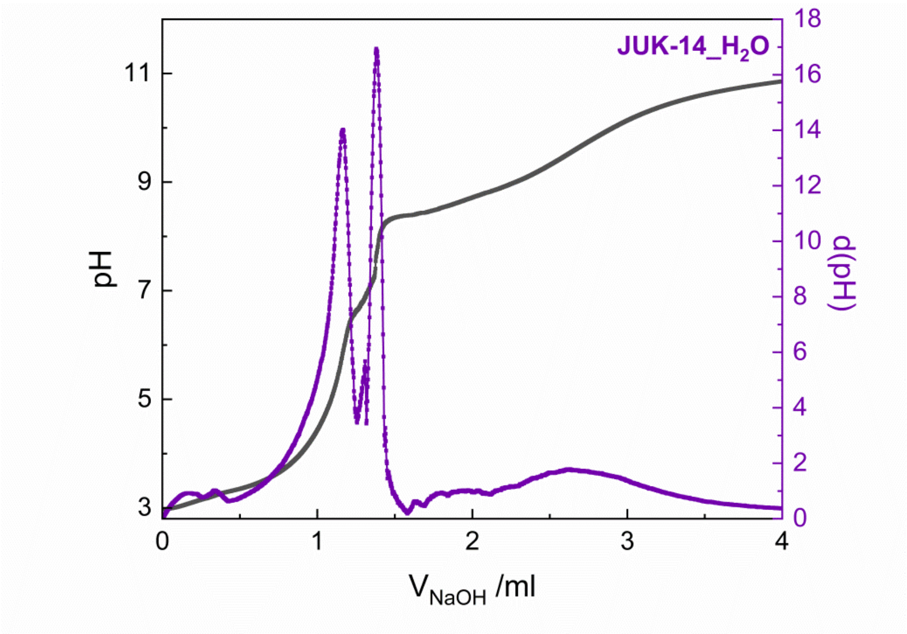

Figure S15. Acid–base titration curve of JUK-14_H_2O (black) and its first derivative curve (violet).

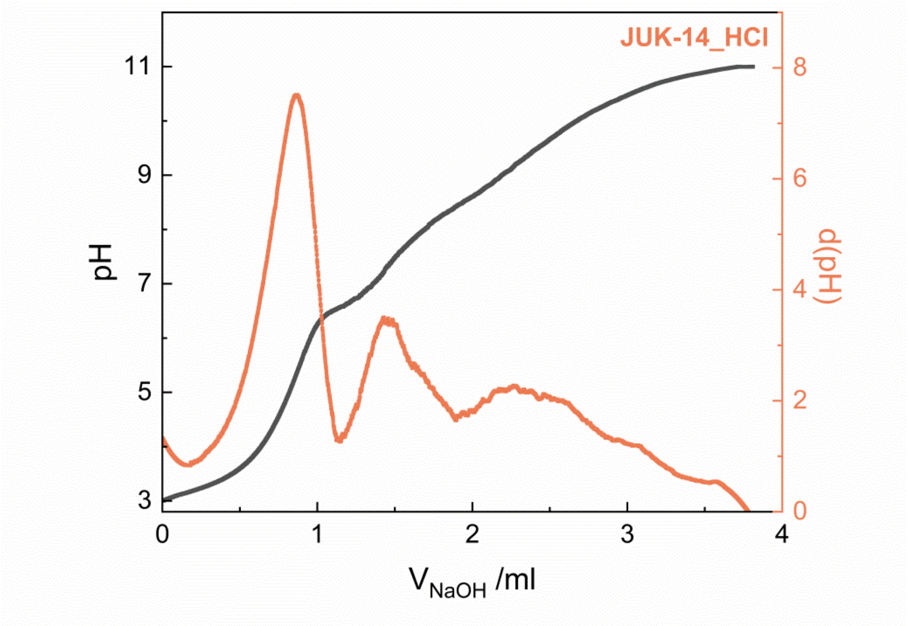

Figure S16. Acid–base titration curve of JUK-14_HCl (black) and its first derivative curve (orange).

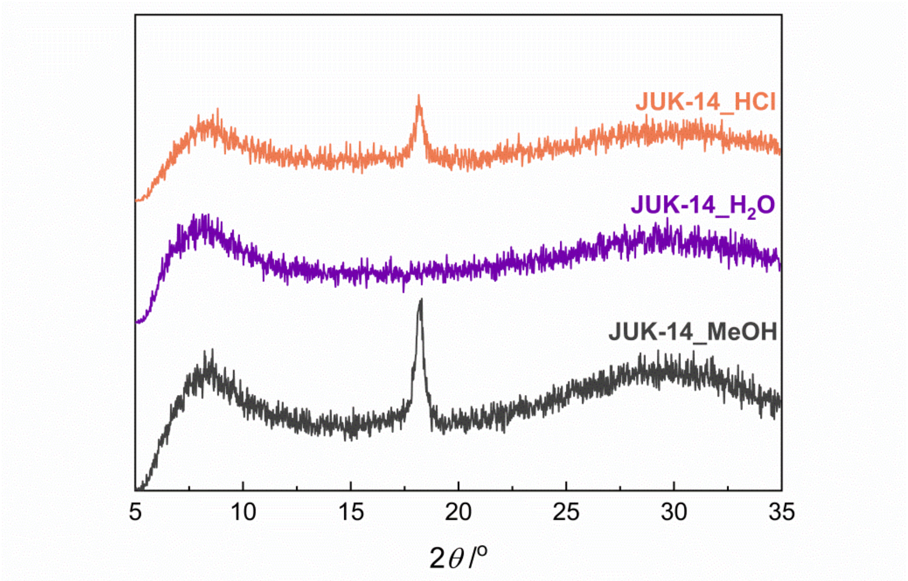

Figure S17. PXRD patterns of JUK-14_MeOH, JUK-14_H_2O and JUK-14_HCl after titration to pH=11.

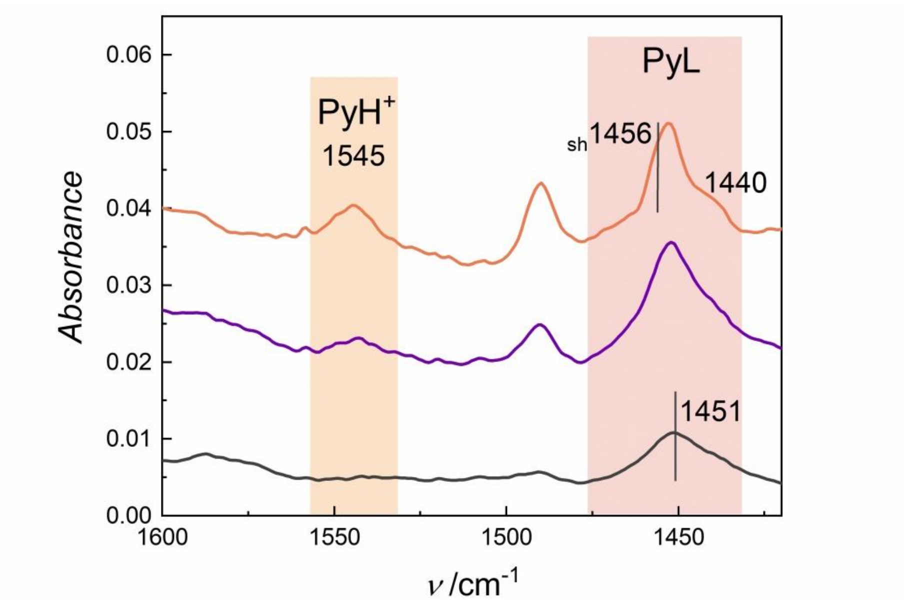

Figure S18. IR differential plots for JUK-14_MeOH (grey), JUK-14_H_2O (violet) and JUK-14_HCl (orange) during the adsorption of pyridine.

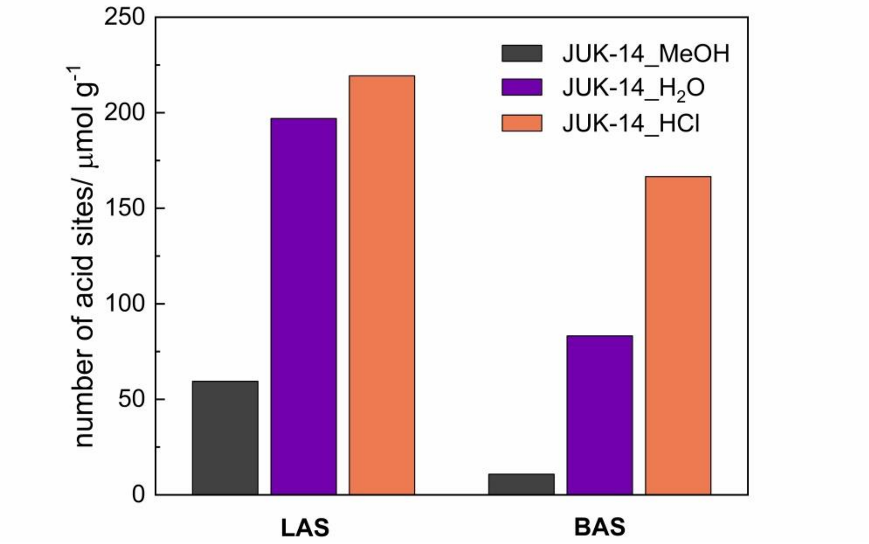

Figure S19. Number of Lewis acid sites (LAS) and Brønsted acid sites (BAS) for samples JUK-14_MeOH, JUK-14_H_2O and JUK-14_HCl. The number of acid centers was calculated per gram of activated sample.

B. Sorption properties of JUK-14 derivatives

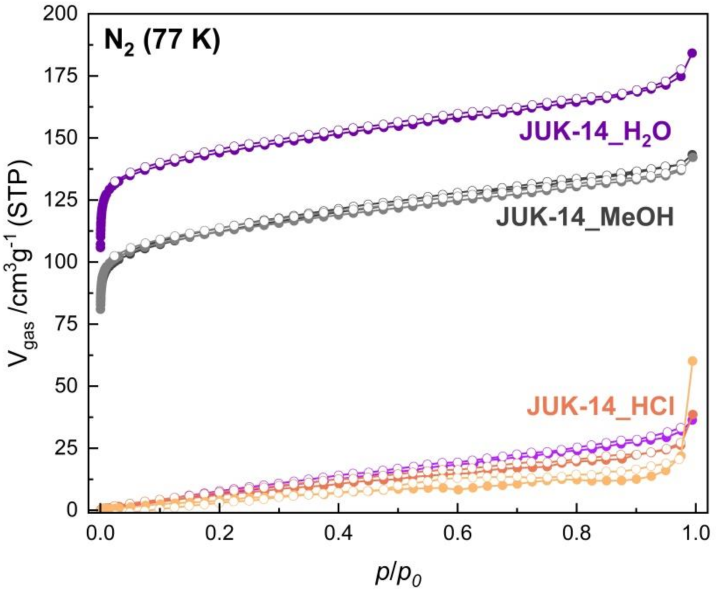

Figure S20. N_2 physisorption isotherms (at 77 K) for JUK-14_MeOH, JUK-14_H_2O and JUK-14_HCl. Solid and open symbols represent adsorption and desorption branches, respectively. Color code is the same as in the main text (Figure 3).

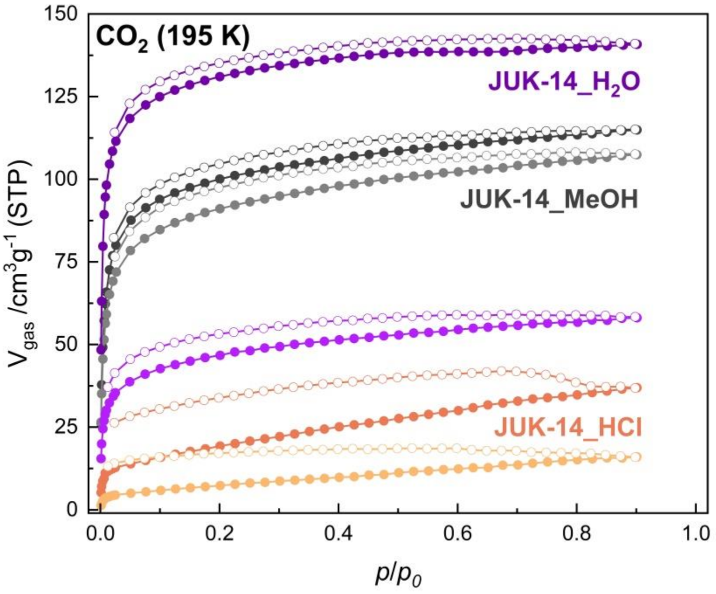

Figure S21. CO_2 physisorption isotherms (at 195 K) for JUK-14_MeOH, JUK-14_H_2O and JUK-14_HCl. Solid and open symbols represent adsorption and desorption branches, respectively. Color code is the same as in the main text (Figure 3).

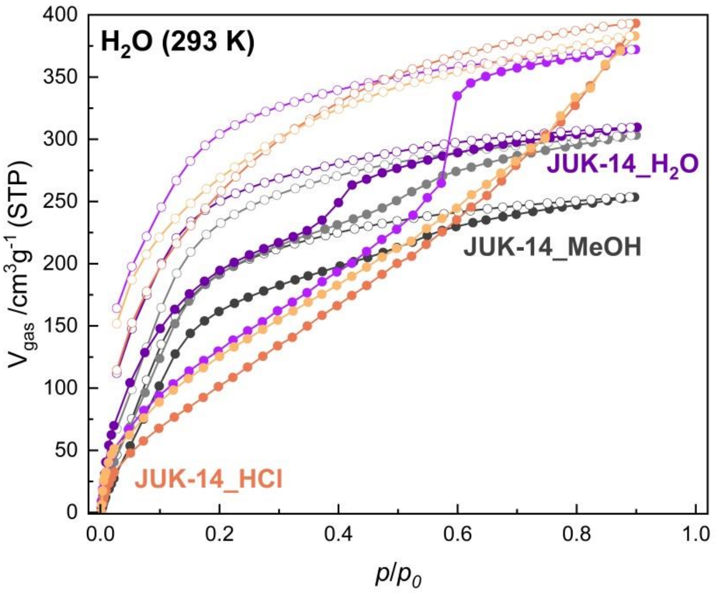

Figure S22. H_2O physisorption isotherms (at 293 K) for JUK-14_MeOH, JUK-14_H_2O and JUK-14_HCl. Solid and open symbols represent adsorption and desorption branches, respectively. Color code is the same as in the main text (Figure 3).

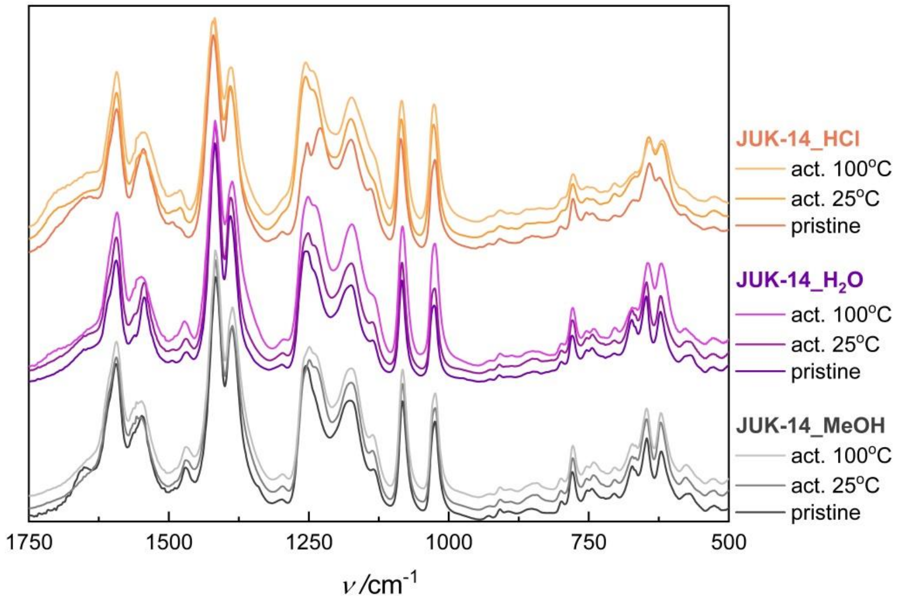

Figure S23. Comparison of IR spectra for JUK-14_MeOH, JUK-14_H_2O and JUK-14_HCl: for pristine materials and for samples after activation under vacuum in 25 and 100°C (*ex situ* measurements).

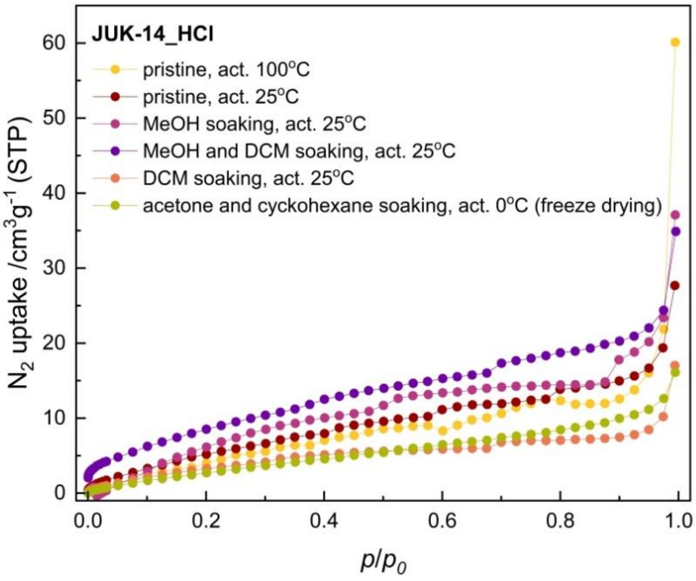

Figure S24. N_2 adsorption isotherms (at 77 K) for JUK-14_HCl after activation by various methods (specified on figure legend). All activations were performed under vacuum. Desorption branches were omitted for clarity.

C. Proton conduction properties of JUK-14 derivatives

For the measurements under controlled humidity conditions, an analysis of measurement uncertainties for activation energies (E_a) and conductivity values (σ) was carried out:

1) The measurement uncertainty of the activation energy was determined by multiplying the derivative of the activation energy with respect to the slope from the Arrhenius plot and the uncertainty of the determination of this slope:

$$\Delta E_a = \left(\frac{\partial E_a}{\partial a}\right)\Delta a = |1000 \cdot k| \cdot \Delta a$$

where: E_a – activation energy,
a – slope; Δa differs for each Arrhenius plot,
k – Boltzmann constant.

2) The measurement uncertainty of the conductivity values was determined as the sum of partial derivatives of the conductivity with respect to the sample thickness, resistance and pellet radius, multiplied by the uncertainties of the corresponding variables:

$$\Delta\sigma = \left(\frac{\partial \sigma}{\partial L}\right)\Delta L + \left(\frac{\partial \sigma}{\partial R}\right)\Delta R + \left(\frac{\partial \sigma}{\partial r}\right)\Delta r$$

$$\Delta\sigma = \left|\frac{1}{\pi R r^2}\right|\Delta L + \left|\frac{-L}{\pi R^2 r^2}\right|\Delta R + \left|\frac{-2L}{\pi R r^3}\right|\Delta r$$

where: σ – proton conductivity,
L – sample thickness; $\Delta L = 0.001\ cm$,
r – pellet radius; $\Delta r = 0.01\ cm$,
R – the resistance as the real part of the measured impedance; The ΔR has been calculated according to the specification of the instrument, provided by the producer,

and varies depending on the resistance value and the frequency range over which the resistance has been measured. Overall, the average percentage ΔR was 3.5% of R.

The calculated measurement uncertainties were presented as error bars in Figure 4 in the main text.

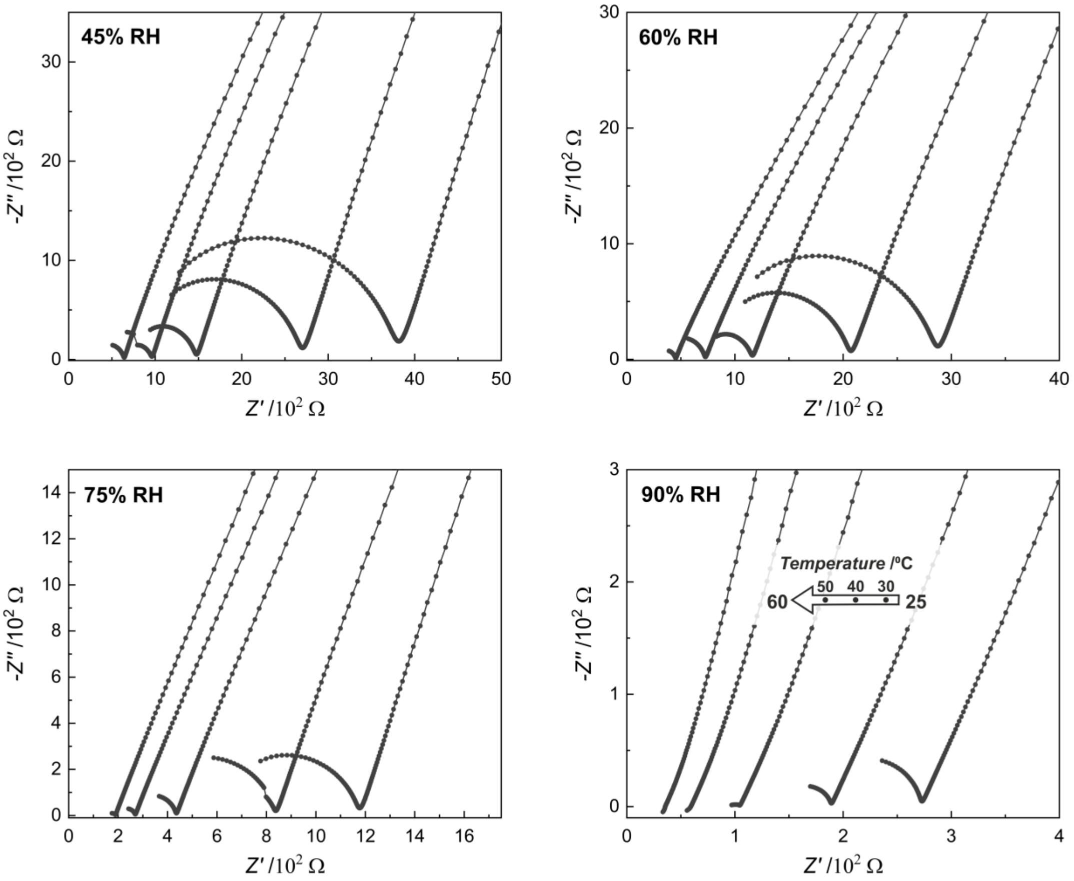

Figure S25. Temperature-dependent impedance plots for JUK-14_MeOH at 45, 60, 75 and 90% RH (specified in the plots). Temperature legend refers to all the plots.

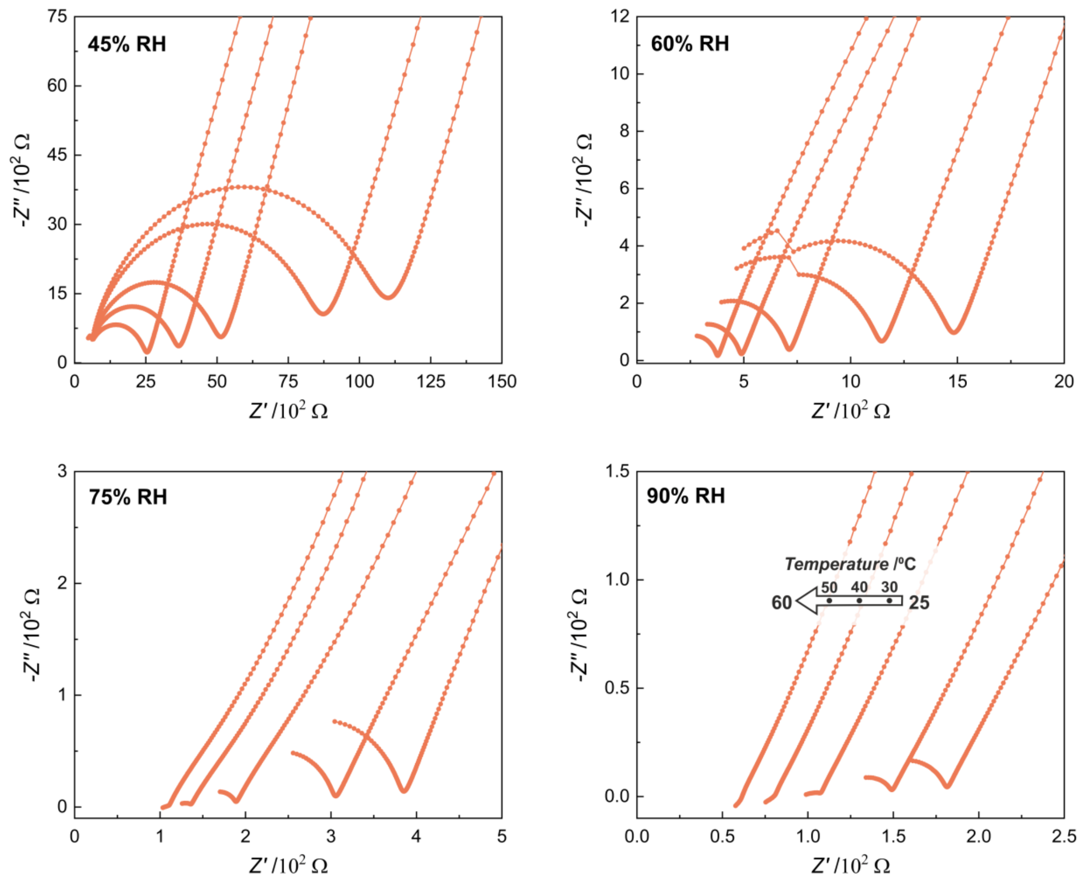

Figure S26. Temperature-dependent impedance plots for JUK-14_HCl at 45, 60, 75 and 90% RH (specified in the plots). Temperature legend refers to all the plots.

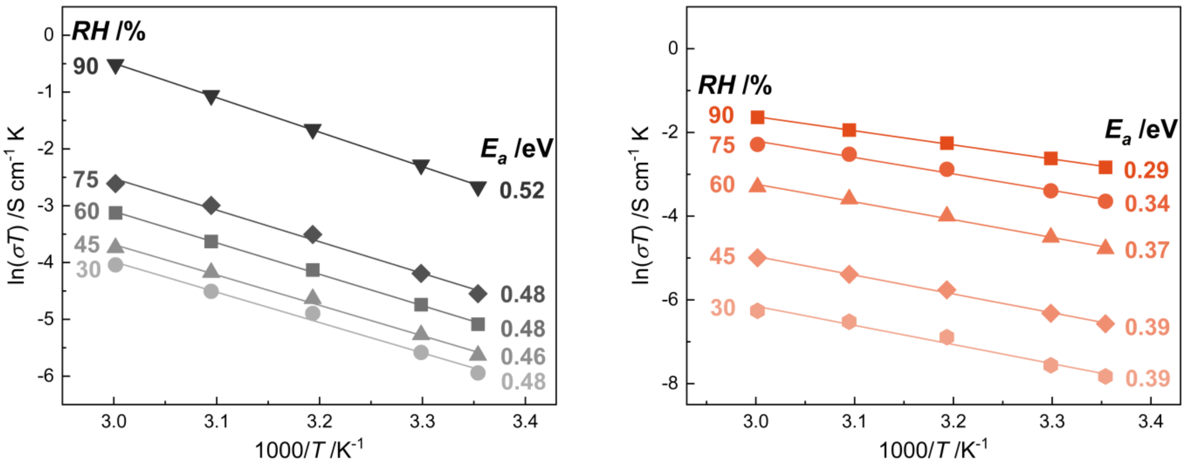

Figure S27. Arrhenius plots with activation energies (E_a) for JUK-14_MeOH (left) and JUK-14_HCl (right) for various RH values in the 25-60ºC range.

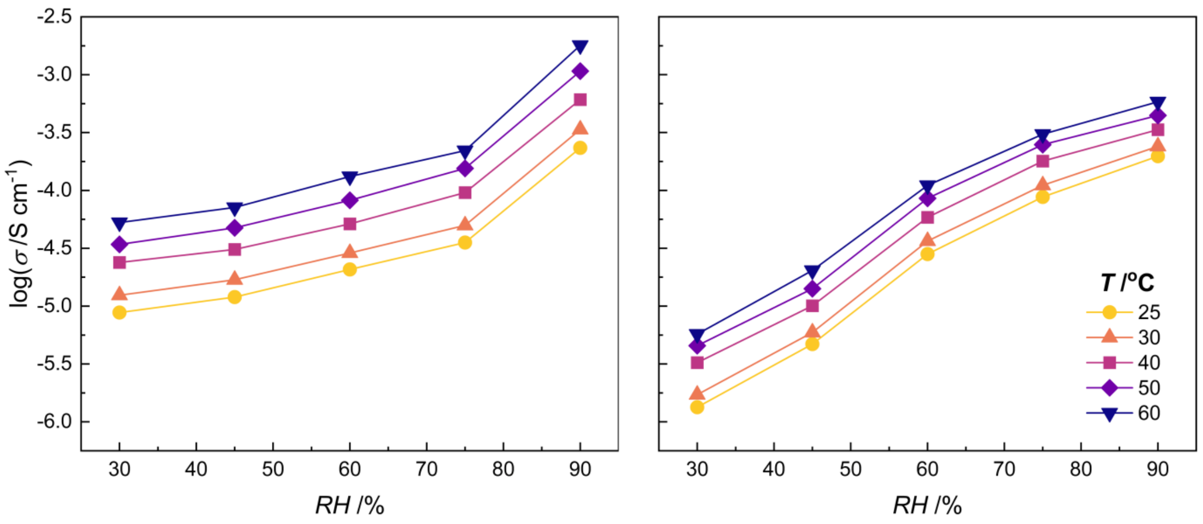

Figure S28. Dependence of proton conductivity on relative humidity for JUK-14_MeOH (left) and JUK-14_HCl (right). Color legend refers to all both plots.

Table S6. Comparison of proton conductivities for JUK-14_MeOH and JUK-14_HCl measured at humid conditions. The results for JUK-14_H_2O was derived from ref. [8].

RH /%	Sample	Conductivity /S·cm^{-1}					Activation energy /eV
		25 °C	30 °C	40 °C	50 °C	60 °C	
30	MeOH	8.8×10^{-6}	1.2×10^{-5}	2.4×10^{-5}	3.4×10^{-5}	5.3×10^{-5}	0.46
	H_2O	1.0×10^{-6}	1.6×10^{-6}	3.7×10^{-6}	5.9×10^{-6}	8.2×10^{-6}	0.54
	HCl	1.3×10^{-6}	1.7×10^{-6}	3.3×10^{-6}	4.6×10^{-6}	5.7×10^{-6}	0.39
45	MeOH	1.2×10^{-5}	1.7×10^{-5}	3.1×10^{-5}	4.8×10^{-5}	7.1×10^{-5}	0.46
	H_2O	3.5×10^{-6}	5.1×10^{-6}	1.0×10^{-5}	1.8×10^{-5}	2.4×10^{-5}	0.51
	HCl	4.7×10^{-6}	5.9×10^{-6}	1.0×10^{-5}	1.4×10^{-5}	2.0×10^{-5}	0.39
60	MeOH	2.1×10^{-5}	2.9×10^{-5}	5.1×10^{-5}	8.2×10^{-5}	1.3×10^{-4}	0.48
	H_2O	7.4×10^{-6}	1.0×10^{-5}	1.9×10^{-5}	3.7×10^{-5}	5.2×10^{-5}	0.52
	HCl	2.8×10^{-5}	3.6×10^{-5}	5.9×10^{-5}	8.5×10^{-5}	1.1×10^{-4}	0.37
75	MeOH	3.5×10^{-5}	5.0×10^{-5}	9.6×10^{-5}	1.5×10^{-4}	2.2×10^{-4}	0.48
	H_2O	2.1×10^{-5}	2.8×10^{-5}	5.5×10^{-5}	8.9×10^{-5}	1.3×10^{-4}	0.48
	HCl	8.8×10^{-5}	1.1×10^{-4}	1.8×10^{-4}	2.4×10^{-4}	3.1×10^{-4}	0.34
90	MeOH	2.3×10^{-4}	3.4×10^{-4}	6.1×10^{-4}	1.1×10^{-3}	1.8×10^{-3}	0.52
	H_2O	5.6×10^{-5}	1.2×10^{-4}	1.7×10^{-4}	2.5×10^{-4}	3.6×10^{-4}	0.36
	HCl	2.0×10^{-4}	2.4×10^{-4}	3.4×10^{-4}	4.4×10^{-4}	5.8×10^{-4}	0.29

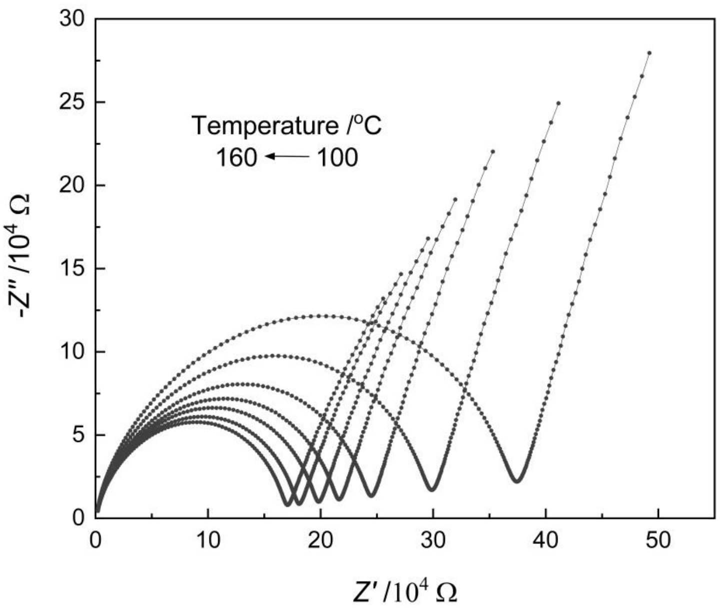

Figure S29. Temperature-dependent impedance plots for JUK-14_MeOH at anhydrous conditions (temperature range 100 – 160 °C).

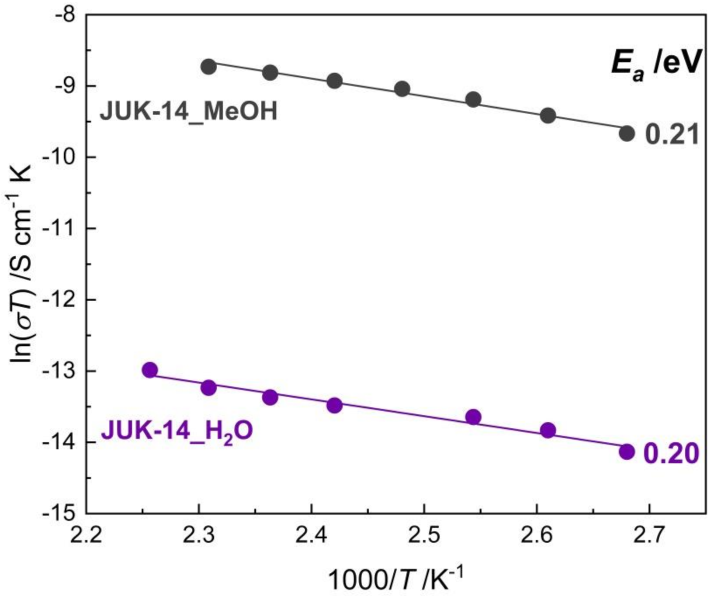

Figure S30. Arrhenius plot with activation energy (E_a) for JUK-14_MeOH in the 100-160°C range. Plot for JUK-14_H_2O was derived from ref. [8] and presented for comparison.

Table S7. List of proton conductivities for JUK-14_MeOH measured at anhydrous conditions. The results for JUK-14_H_2O was derived from ref. [8].

Sample	Conductivity /S·cm^{-1}							Activation energy /eV
	100 °C	110 °C	120 °C	130 °C	140 °C	150 °C	160 °C	
**JUK-14_MeOH**	1.7×10^{-7}	2.1×10^{-7}	2.6×10^{-7}	2.9×10^{-7}	3.2×10^{-7}	3.5×10^{-7}	3.7×10^{-7}	0.21
**JUK-14_H_2O**	2.0×10^{-9}	2.6×10^{-9}	3.0×10^{-9}	-	3.4×10^{-9}	3.7×10^{-9}	4.1×10^{-9}	0.20

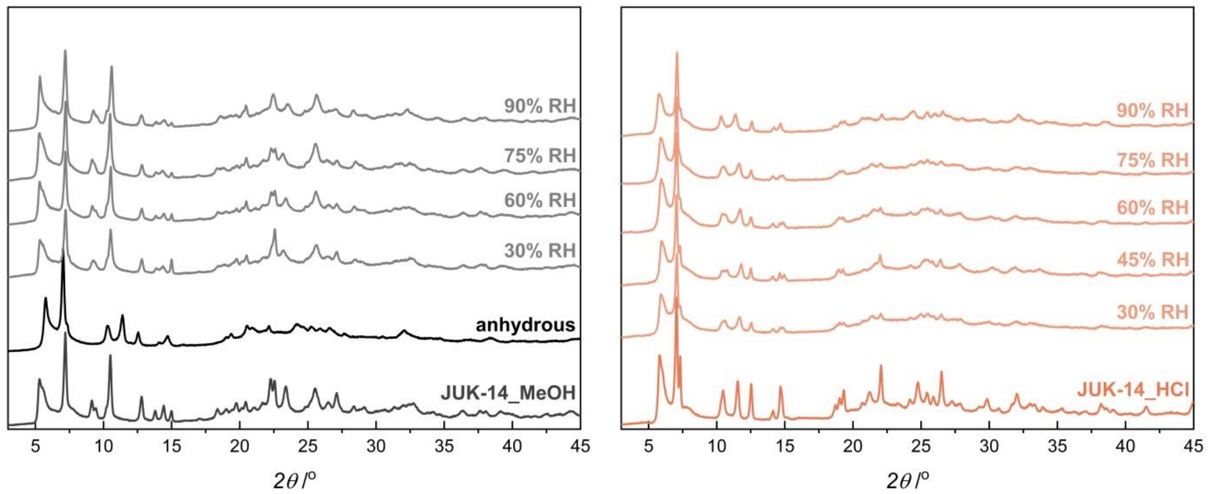

Figure S31. PXRD patterns of JUK-14_MeOH (left) and JUK-14_HCl (right) after EIS measurements under specified relative humidity and under anhydrous conditions (JUK-14_MeOH) compared with PXRD patterns of the corresponding materials before EIS measurements.

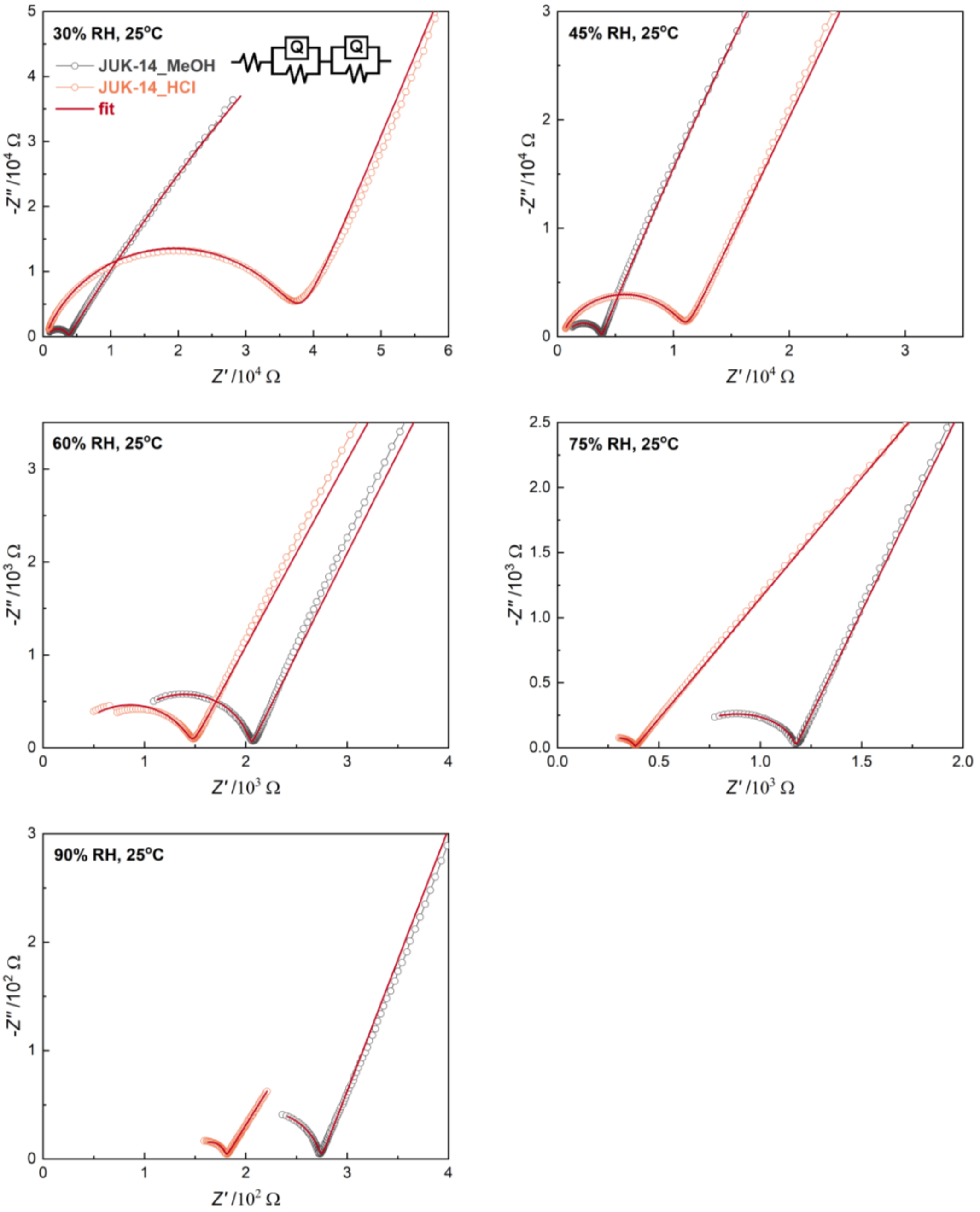

Figure S32. Impedance plots for JUK-14_MeOH (grey) and JUK-14_HCl (orange) at 25 ºC and different relative humidity; open circles – measured data, red line – fitted curves (with the use of ZSimpWin software). The equivalent circuit used for fitting the AC impedance plots was depicted on the first graph and refers to all the plots.

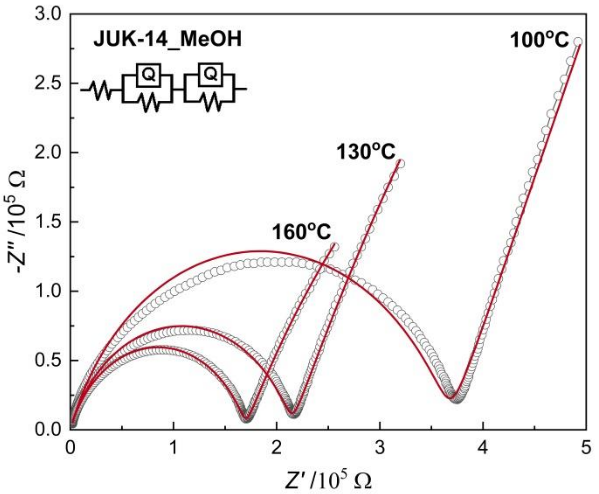

Figure S33. Impedance plots for JUK-14_MeOH at 100, 130 and 160 °C; black circles – measured data, red line – fitted curves (with the use of ZSimpWin software). The equivalent circuit used for fitting the AC impedance plots was depicted on the graph.

References

(1) Taylor, J. M.; Dekura, S.; Ikeda, R.; Kitagawa, H. Defect Control To Enhance Proton Conductivity in a Metal–Organic Framework. *Chem. Mater.* **2015**, *27* (7), 2286–2289. https://doi.org/10.1021/acs.chemmater.5b00665.

(2) Taylor, J. M.; Komatsu, T.; Dekura, S.; Otsubo, K.; Takata, M.; Kitagawa, H. The Role of a Three Dimensionally Ordered Defect Sublattice on the Acidity of a Sulfonated Metal–Organic Framework. *J. Am. Chem. Soc.* **2015**, *137* (35), 11498–11506. https://doi.org/10.1021/jacs.5b07267.

(3) Liu, Q.-Q.; Liu, S.-S.; Liu, X.-F.; Xu, X.-J.; Dong, X.-Y.; Zhang, H.-J.; Zang, S.-Q. Superprotonic Conductivity of UiO-66 with Missing-Linker Defects in Aqua-Ammonia Vapor. *Inorg. Chem.* **2022**, *61* (8), 3406–3411. https://doi.org/10.1021/acs.inorgchem.1c03231.

(4) Basu, O.; Mukhopadhyay, S.; Laha, S.; Das, S. K. Defect Engineering in a Metal–Organic Framework System to Achieve Super-Protonic Conductivity. *Chem. Mater.* **2022**, *34* (15), 6734–6743. https://doi.org/10.1021/acs.chemmater.2c00654.

(5) He, Y.; Dong, J.; Liu, Z.; Li, M.-Q.; Hu, J.; Zhou, Y.; Xu, Z.; He, J. Dense Dithiolene Units on Metal–Organic Frameworks for Mercury Removal and Superprotonic Conduction. *ACS Appl. Mater. Interfaces* **2022**, *14* (1), 1070–1076. https://doi.org/10.1021/acsami.1c20762.

(6) Jagiełło, J.; Bandosz, T. J.; Schwarz, J. A. Carbon Surface Characterization in Terms of Its Acidity Constant Distribution. *Carbon* **1994**, *32* (5), 1026–1028. https://doi.org/10.1016/0008-6223(94)90066-3.

(7) Yashchenko, V. S.; Pap, A. A.; Kalechits, G. V.; Makey, A. V.; Ol'khovik, V. K. Synthesis of 10,10-Dioxo-10λ6-Phenoxathiin Derivatives by Reaction of 4,4'-Oxydibenzoic Acid with Sulfur-Containing Electrophiles*. *Chem Heterocycl Comp* **2015**, *50* (10), 1471–1477. https://doi.org/10.1007/s10593-014-1612-2.

(8) Szufla, M.; Choroś, A.; Nitek, W.; Matoga, D. A Porous Sulfonated 2D Zirconium Metal–Organic Framework as a Robust Platform for Proton Conduction. *Chemistry A European J* **2022**, *28* (37). https://doi.org/10.1002/chem.202200835.
